# Bicontinuous vitrimer heterogels with wide-span switchable stiffness-gated iontronic coordination

**DOI:** 10.1126/sciadv.adl2737

**Published:** 2024-03-08

**Authors:** Ziguang Zhao, Ziquan Cao, Zhixin Wu, Wenxin Du, Xue Meng, Huawei Chen, Yuchen Wu, Lei Jiang, Mingjie Liu

**Affiliations:** ^1^ School of Future Technology, University of Chinese Academy of Sciences, Beijing 100190, P. R. China.; ^2^ Key Laboratory of Bio-Inspired Materials and Interfacial Science, Technical Institute of Physics and Chemistry, Chinese Academy of Sciences, Beijing 100190, P. R. China.; ^3^ School of Mechanical Engineering and Automation, Beihang University, Beijing 100191, China.; ^4^ Key Laboratory of Bio-Inspired Smart Interfacial, Science and Technology of Ministry of Education, School of Chemistry, Beihang University, Beijing 100191, P.R. China.

## Abstract

Currently, it remains challenging to balance intrinsic stiffness with programmability in most vitrimers. Simultaneously, coordinating materials with gel-like iontronic properties for intrinsic ion transmission while maintaining vitrimer programmable features remains underexplored. Here, we introduce a phase-engineering strategy to fabricate bicontinuous vitrimer heterogel (VHG) materials. Such VHGs exhibited high mechanical strength, with an elastic modulus of up to 116 MPa, a high strain performance exceeding 1000%, and a switchable stiffness ratio surpassing 5 × 10^3^. Moreover, highly programmable reprocessing and shape memory morphing were realized owing to the ion liquid–enhanced VHG network reconfiguration. Derived from the ion transmission pathway in the ILgel, which responded to the wide-span switchable mechanics, the VHG iontronics had a unique bidirectional stiffness-gated piezoresistivity, coordinating both positive and negative piezoresistive properties. Our findings indicate that the VHG system can act as a foundational material in various promising applications, including smart sensors, soft machines, and bioelectronics.

## Teaser

Vitrimer gels with bicontinuous heterostructures have the wide-span switchable stiffness-gated iontronic coordination.

## INTRODUCTION

Programmable vitrimers are a promising type of smart dynamic polymer materials that have attracted considerable attention in recent years owing to their unique properties (*1*–*4*). The adaptive dynamic covalent networks of vitrimers exhibit programmable features, including switchable mechanics, shape memory, self-healing, and reprocessability, which are not typically found in conventional polymer and gel materials (*5*–*8*). The chemical network design of programmable vitrimers generally depends primarily on switchable polymer segments (e.g., glass transition or melting cyclization) and activated bond exchange netpoints (*9*, *10*). Under specific conditions, typically influenced by thermal effects or catalysts, dynamic chemical reactions can trigger the vitrimer network’s reconfiguration (*11*, *12*). However, owing to the high density of cross-linking netpoints and the low flexibility of polymer segments, it remains challenging to balance intrinsic stiffness with programmability in most vitrimer materials, limiting their broader application. For example, the high-strain capacity (with a strain ratio exceeding 1000%) and the wide-span switchable mechanics (with a stiffness ratio exceeding 10^3^) still cannot be simultaneously coordinated (*13*, *14*). Although introducing the solvation effect to the vitrimer network enabled the construction of a high-strain soft gel (*15*, *16*), it conversely weakened the switchable polymer segments’ transition mechanism, thereby diminishing their programmable properties. Furthermore, compared with current quasi-solid gel materials (*17*–*20*), vitrimers should have made more advancements in the fields of smart sensors, energy storage, and iontronics (*21*). Along with their programmable features, coordinating vitrimers with gel-like iontronic properties for intrinsic ion transmission and exchange remains a challenge. Therefore, here, we propose a phase-engineering strategy for vitrimer materials to effectively address these limitations. The bicontinuous structure, as a crucial architecture, integrates two distinct interpenetrating frameworks to synergistically realize the optimization of the materials’ programmable properties and functional coordination.

In our study, derived from the phase coordination of a stiff vitrimer and soft ion-liquid gel (ILgel), each acting as individual continuous phase frameworks, the bicontinuous vitrimer heterogel (VHG) materials realized high mechanical performance and a wide-span switchable stiffness feature. The maximal ratio of switchable stiffness in VHG systems could surpass 5 × 10^3^, in which a high-strain deformation of more than 1000% and an associated elastic modulus of up to 116 MPa were exhibited. Compared with conventional vitrimer systems that use extra non-green catalysts (*22*, *23*), our approach introduces specific ion liquids that act as the ILgel dispersion phase to enhance the VHG’s vitrimer network reconfiguration, resulting in highly programmable reprocessing and shape memory effects. Thus, complex origami and kirigami shape memory morphings could also be realized. Unlike existing piezoresistive systems with unidirectional negative/positive features (*24*, *25*), the VHG iontronics demonstrated the bidirectional stiffness ion gating, coordinately integrating negative and positive piezoresistive signals to greatly enhance the sensor’s sophisticated awareness capabilities. Such an extension of the bicontinuous VHG architecture to iontronic systems can open up intriguing opportunities to construct ionic or molecular variational transport mechanisms. We expect that such bicontinuous structures and functionalities of VHG systems will also provide a phase-engineering technology for the design of highly programmable vitrimer and gel architectures that can be implemented in the material foundations of smart sensors, soft machines, and energy storage devices.

## RESULTS

### Design and structural feature of bicontinuous VHG heterogels

**Fig. 1.**
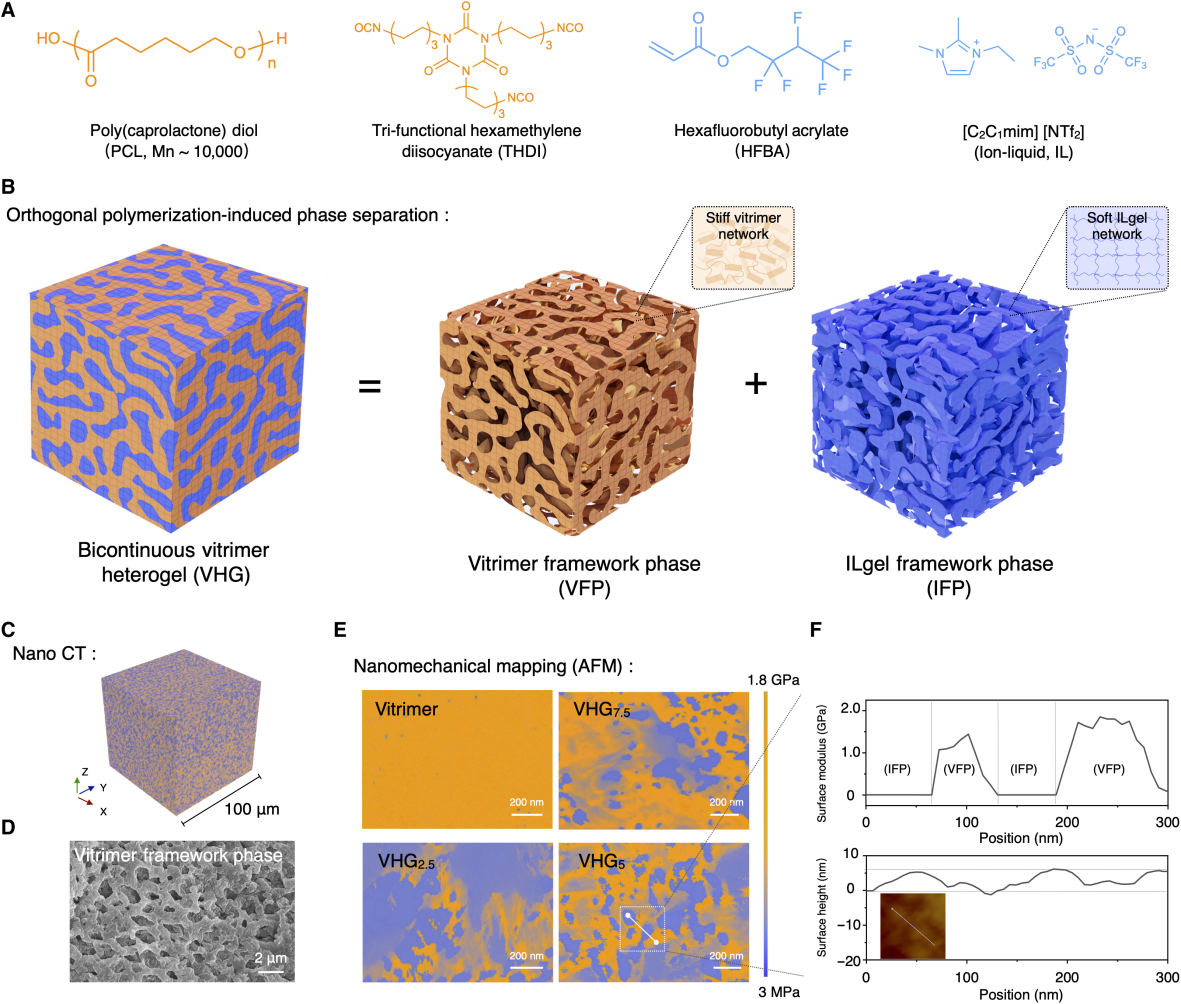
Design and structural feature of bicontinuous vitrimer heterogels. (**A**) Chemical structures of reacted precursor components within VHGs. (**B**) VHGs have the bicontinuous structure of vitrimer (VFP) and ILgel (IFP) framework phases owing to the orthogonal polymerization-induced phase separation. (**C**) Nano CT image demonstrating the bicontinuous phase structure of VHG_5_. Two distinct VFP (orange) and IFP (blue) domains seamlessly interpenetrated, with each phase domain forming a continuous path. (**D**) SEM image demonstrating the continuous morphologies of the VFP network in VHG following the removal of the ILgel phase. (**E**) AFM nanomechanical mapping revealed the bicontinuous morphology of VHGs and exhibited distinct mechanical properties between VFP and IFP. (**F**) Surface modulus and surface height variation of VHG_5_.

In this work, we used an orthogonal polymerization-induced phase separation strategy to fabricate VHG materials featuring bicontinuous architectures. Figure 1A illustrated the chemically reacted precursor components of VHGs. Initially, these precursors were combined to yield a clear and homogeneous liquid system. During the orthogonal reaction process, the formation of the heterogeneous vitrimer gelation could be distinctly observed (fig. S1A). Within VHGs, the vitrimer framework phase (VFP) referred to a polyurethane reaction between poly(caprolactone) diol (PCL-diol) and tri-functional hexamethylene diisocyanate (THDI), and the ILgel framework phases (IFPs) derived from an ultraviolet (UV) free-radical polymerization of hexafluorobutyl acrylate (HFBA) (Fig. 1B). The rheological test demonstrated that the VHG’s gelation point rapidly occurred through simultaneously applied UV light and heat conditions (fig. S1B). As such a “one-step” orthogonal polymerization process, the VFP and IFP components underwent the phase separation process, leading to the gradual formation of the VHG bicontinuous structure. This was attributed to the dynamic equilibrium established by the biphasic interfacial tension and the associated hybrid entropy. In contrast to conventional dibutyltin dilaurate (DBTDL)–catalyzed vitrimer systems, our VFP construction eliminated the need for such catalysts. Instead, the ion liquid ([C_2_C_1_mim] [NTf_2_]) acted as an interfacial catalyst to expedite the related polyurethane reaction, which was reflected by the rheological properties of the IL-PCL vitrimer (fig. S1C). Furthermore, fig. S2 exhibited the macroscopic feature of phase separation in VHG. Owing to the melting crystallization property, the PCL vitrimer switched to a transparent state above its melting point (*T* > *T*_m_). VHGs maintained an opaque state regardless of whether the temperature was above *T*_m_ or below *T*_m_. The thermal analysis results show a consistent phase transition between the PCL vitrimer and VHG (fig. S3). These results indicate that the VFP component, constituting a stable individual phase within VHG, cannot be affected by the solvation effect of the IFP component.

To further confirm the bicontinuous phase morphology of VHGs at the microscopical level, we used nano-computed tomography (Nano CT) scanning and atomic force microscopy (AFM) measurements. The density distinction between the PCL vitrimer and the ILgel allowed the biphasic VHG structure to be visualized through the associated density distribution, as measured by x-ray absorption in Nano CT. Figure 1C illustrates that two distinct VFP and IFP domains were seamlessly interpenetrated, with each phase domain forming a continuous path throughout the VHG system. Owing to the solvating effect of ethanol, we could selectively remove the ILgel phase from VHG networks to isolate the VFP network, and the scanning electron microscopy (SEM) image demonstrated the VFP’s continuous phase morphologies within the VHG materials (Fig. 1D). The nanomechanical mapping results obtained from AFM revealed the bicontinuous structure by distinct mechanical properties between VFP and IFP (Fig. 1E). The orange-labeled VFP and PCL vitrimer exhibited a notably high surface modulus of 1.8 GPa owing to the PCL network’s stiff crystalline feature. In contrast, the blue-labeled IFP with its inherent softness displayed an extremely low surface modulus of 3 MPa. By modulating the phase proportion in VHG systems, we could also observe different bicontinuous phase morphologies. These samples were denoted as VHG*_x_*, where *x* represents the weight ratio of VFP to VHG (which was fixed to 10). Despite variations in the proportion of the two phases, both were continuous and intertwined in VHG systems. The nanomechanical mapping of VHG_7.5_ demonstrated that the IFP permeated and was embedded in the VFP matrix. In addition, in VHG_5_, we also found a uniform bicontinuous structure where VFP occupied approximately 50% of the volume, with the morphological cross-sectional size of the dual phases ranging from microscale to nanoscale. Figure 1F illustrates the surface modulus variation of VHG_5_, ranging from 1.8 GPa and 3.5 MPa in a flat region with fluctuations within a 10-nm range, indicating a bicontinuous morphological distribution of the VFP and IFP components. Moreover, swelling-deswelling tests were conducted for the VFP network and the PCL vitrimer, further confirming their stable cross-linked structure (fig. S5).

### High mechanical performance and wide-span switchable stiffness

**Fig. 2.**
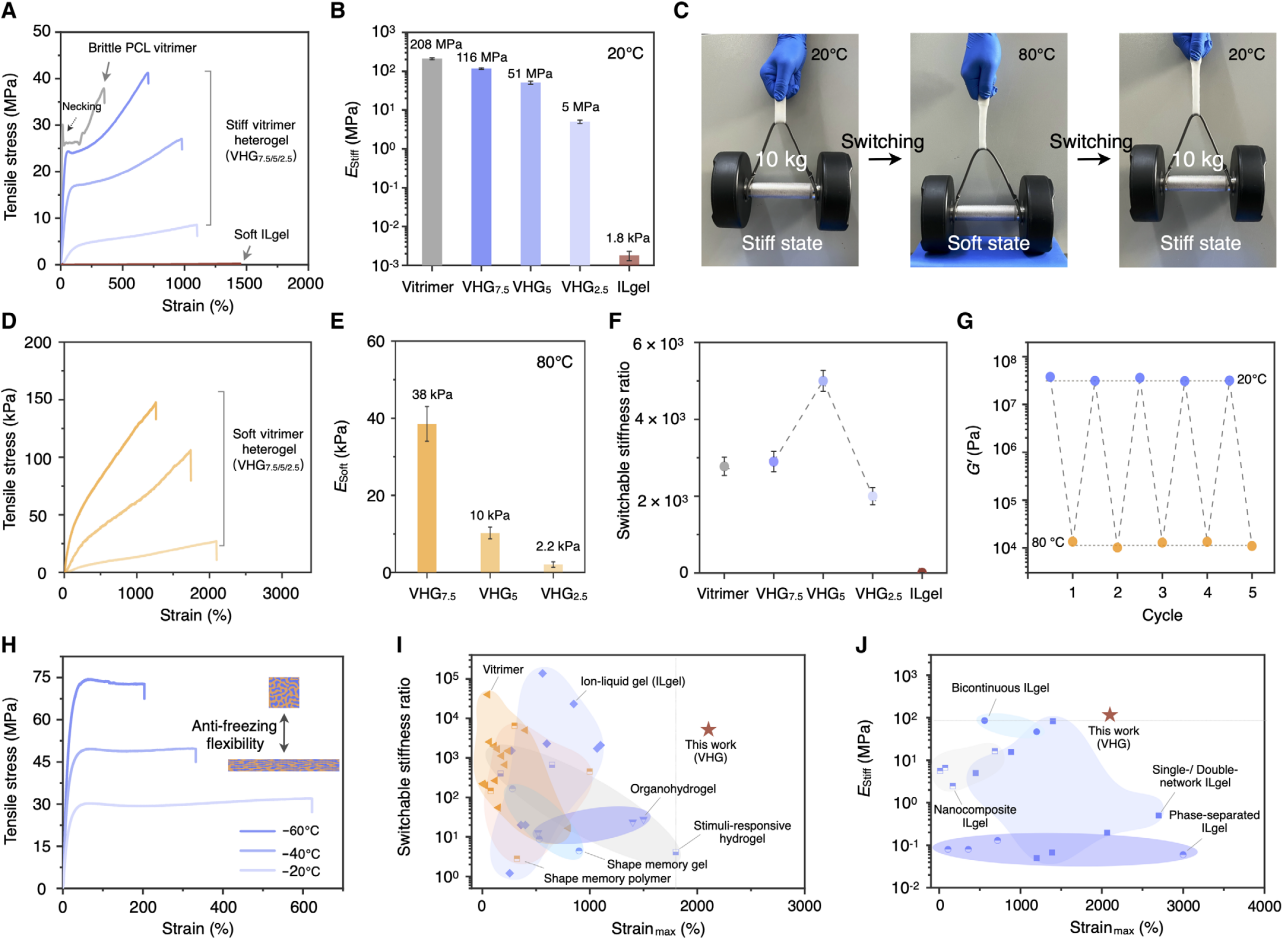
High mechanical performance and wide-span switchable stiffness. (**A** and **B**) Tensile stress-strain curves and elastic modulus (*E*_stiff_) of the brittle PCL vitrimer, the soft ILgel, and VHGs with different VFP and IFP components at 20°C, respectively. (**C**) VHG_7.5_ with a thickness of 1 mm can easily withstood a tensile load of 10 kg, even when undergoing temporary thermoplastic deformation. (**D** and **E**) Tensile stress-strain curves and elastic modulus (*E*_soft_) at 80°C for the different VHGs. (**F**) Switchable stiffness ratios of the PCL vitrimer, the ILgel, and the different VHGs, respectively. (**G**) Stable transitions between high and low storage modulus (*G*′) of VHG_5_ at 20° and 80°C. (**H**) Tensile stress-strain curves of VHG_5_ under cooling conditions (−20°, −40°, and −60°C). (**I**) Analysis comparing the switchable stiffness ratio to strain_max_ in VHGs with that of existing polymer materials with switchable mechanics, including vitrimers, shape memory polymers and gels, organohydrogels, stimuli-responsive gels, and ILgels. (**J**) Comparative analysis of VHGs and other typical ILgels with different structures (i.e., single/double network, nanocomposite, phase-separated network, and bicontinuous network), focusing on the relationship between *E*_stiff_ and strain_max_.

The VHGs’ mechanical reinforcement performance was predominantly attributed to the coordination effects of the bicontinuous phases and interfaces, which enabled the material to achieve high mechanical properties when subjected to external stresses. The PCL vitrimer had high mechanical stiffness, with an elastic modulus exceeding 200 MPa. Owing to the low flexibility and deformability of a semi-crystalline polymer network, the PCL vitrimer exhibited an obvious necking phenomenon and could not efficiently dissipate excess energy, leading to brittle fractures. Conversely, the ILgel displayed a low elastic modulus of 1.8 kPa and a high stretching ratio of up to 1480%. Integrating the bicontinuous structure with these two distinct mechanical properties, VHGs exhibited both high mechanical strength and stretchability (Fig. 2, A and B). VFP offered strong mechanical support, whereas the IFP predominantly dissipated external energy to enhance the system’s impact resistance and toughness. Moreover, the bicontinuous interface also served as a critical barrier to crack propagation. For example, VHG_7.5_ yielded a stiff elastic modulus (*E*_stiff_) of 116 MPa and could withstand a stretchable deformation up to 600%. In Fig. 2C, the 1-mm-thick VHG_5_ easily withstood a tensile load of 10 kg, even when undergoing a temporary thermoplastic deformation.

Owing to the phase transition property of the PCL vitrimer, the wide-span switchable stiffness feature of VHGs can be activated. In both compressive and tensile tests, the VHG systems displayed enormous variations in mechanical strength and stretchability. VHG_2.5_ had a soft elastic modulus (*E*_soft_) of merely 2.2 kPa at 80°C (*T* > *T*_m_), accompanied by an impressive stretching ratio of up to 2100% (Fig. 2D). In contrast, the PCL vitrimer with the phase transition soft state could not undergo a stretching strain of over 1000% (fig. S6). Figure 2E reveals that the VHGs’ mechanical modulus variation spanned up to three orders of magnitude, in which the VHG_5_’s switchable stiffness ratio (*E*_stiff_/*E*_soft_) notably exceeded 5 × 10^3^ (Fig. 2F). Furthermore, such wide-span switchable mechanical properties were also observed in compressive tests (fig. S7). In the rheological and mechanical measurements, stable transitions between a high and low elastic modulus in VHG systems are reflected in Fig. 2G and figs. S8 and S9. However, we also focused on the mechanical stability of VHGs under cooling conditions. In the bicontinuous framework structure, the ion liquid ([C_2_C_1_mim] [NTf_2_]) demonstrated high environmental adaptability, effectively preserving the solvation effect for the IFP network even at temperatures as low as −60°C, achieving the anti-freezing flexibility of VHG materials to further expand complex conditional applications (Fig. 2H).

Two critical attributes of smart mechanical materials are mechanical properties and switchable mechanics. Achieving high-strain performance remains a challenge in conventional vitrimer materials with switchable mechanics, primarily owing to the inherent limited flexibility originating from stiff polymer networks and high-density netpoints. Moreover, most reported bicontinuous ILgels are fabricated by the phase separation approaches between the polymer network phase and the dispersion phase, which display a high elastic modulus owing to the polymer network phase separation collision. The dissociative dispersion phase in such a phase separation gel system also results in unstable structures and defects. In VHG systems, the bicontinuous architectures between the “softness” and “stiffness” framework phases indicate optimal mechanical coordination. We conducted two comparative analyses to contrast the switching ratios, elastic modulus, and high-strain features in current switchable mechanical polymers and ILgel materials, highlighting the mechanical and structural advantages of bicontinuous VHG systems (Fig. 2, I and J, and tables S1 and S2).

### Ion liquid–enhanced dynamic bicontinuous network reconfiguration

**Fig. 3.**
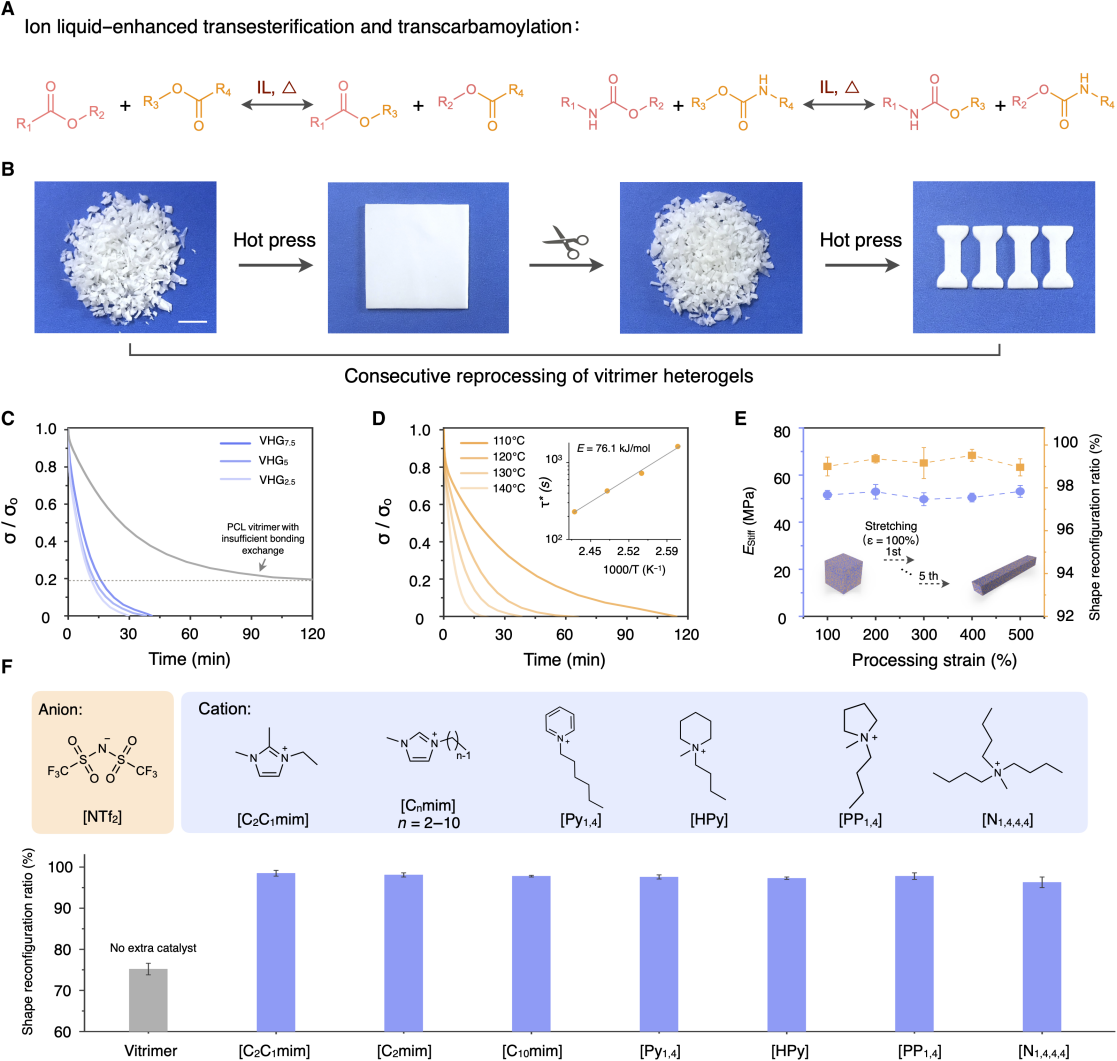
Ion liquid–enhanced dynamic bicontinuous network reconfiguration. (**A**) Schematic illustration of the enhancement of both transesterification and transcarbamoylation processes by ion liquid in VHG systems. (**B**) Consecutive reprocessing of VHG_5_ with a dynamic bicontinuous reconfiguration. (**C**) Stress relaxation behaviors of the PCL vitrimer and VHGs with different VFP and IFP components at 130°C. (**D**) Stress relaxation and Arrhenius analysis of VHG_5_ as the temperature increased from 110° to 140°C. (**E**) *E*_stiff_ and shape reconfiguration ratios of VHG_5_ during consecutive reprocessing, with processing strains progressively increasing from 100 to 500%. (**F**) In VHG systems, various ILs contained [NTf_2_] as the anion enabled the enhancement of bicontinuous network reconfiguration. Scale bar, 1 cm.

Ion liquids serve a dual function in our system—as the IFP dispersion phase and as an interfacial catalyst to enhance the associative dynamic covalent-bonding exchanges, resulting in the bicontinuous network reconfiguration in VHG systems. Unlike conventional polyurethane vitrimers that rely on additional non-green catalysts, the introduction of the ion-liquid phase offers a worthwhile strategy that triggers the reprocessing features of covalent adaptive networks. In each VHG system, we maintained a consistent molar ratio of hydroxyl groups from PCL polymers to the −NCO groups of THDI, approximately 1:0.9. Figure S10 shows the characteristic absorption peaks of the residual hydroxyl groups in the Fourier transform infrared spectrum of the PCL vitrimer. Residual hydroxyl groups were intentionally preserved within the VFP network, which ensured the stability of dynamic covalent bond exchanges. Figure 3A shows that [C_2_C_1_mim] [NTf_2_], as an organic amine ion-liquid catalyst, enhanced both transesterification and transcarbamoylation in the VFP network of the VHG system. In the bicontinuous architectures, the soft IFP was also constrained by the stiffer VFP network. As a result, the consecutive reprocessing of VHG_5_ was achieved through cyclic hot pressing at 130°C (Fig. 3B). Moreover, we conducted a quantitative analysis to assess how the bicontinuous structures and catalyzed temperatures influence the network reconfiguration of VHGs (fig. S11). As shown in Fig. 3C, all VHGs with different bicontinuous phase components could complete their stress relaxation within 40 min at 130°C. Conversely, the catalyst-free PCL vitrimer and VFP network relaxed to no more than 90% even after 120 min, indicating their insufficient bonding exchange (fig. S12A). The creep compliance result of VHG_5_ displayed a thermally adaptive deformation, demonstrating the dynamic network reconfiguration in VHGs (fig. S12B). The ion liquid–catalyzed mechanism was coordinated with the activation temperature of two exchange reactions. As the temperature increased from 110° to 140°C, the stress relaxation time of VHG_5_ correspondingly decreased, and the related Arrhenius kinetics of the network reconfiguration yielded an apparent activation energy (*E*) of 76.1 kJ/mol (Fig. 3D). During consecutive stretching reprocessing, with processing strains progressively increasing from 100 to 500%, *E*_stiff_ of VHG_5_ remained almost consistent, and its shape reconfiguration ratios were all above 98%, exhibiting a stable dynamic bicontinuous network reconfiguration (Fig. 3E and fig. S13). The thermogravimetric analysis results between the original VHG and the VHG sample after stress relaxation are nearly identical, confirming that no thermal degradation occurred during the stress relaxation process (fig. S14). In addition, VHGs also displayed remarkable resistance to yellowing compared with the DBTDL-catalyzed heterogel system (fig. S15). In VHG systems, various ion liquids contained [NTf_2_] as the anion, enhancing the dynamics of the bicontinuous network and demonstrating our strategy’s general applicability (Fig. 3F and table S3). We also found that some other ion liquids were incapable of accelerating the polyurethane reaction to fabricate the VFP network and further construct stable bicontinuous structures for VHG systems (table S4).

### Programmable shape memory morphing property

**Fig. 4.**
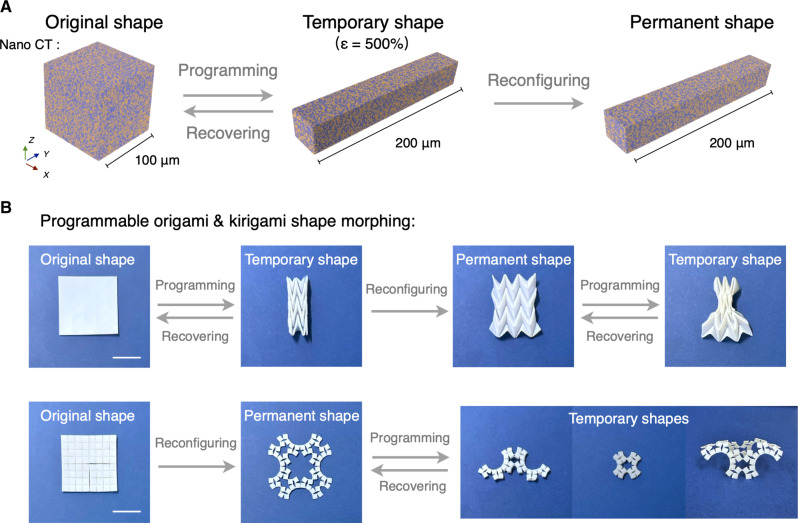
Programmable shape memory morphing property. (**A**) Nano CT images exhibiting variations in the bicontinuous structure of VHG_5_ during the programmable shape memory process. (**B**) Highly programmable shape memory morphings were based on two specific techniques: Miura origami and square-patterned kirigami. Scale bar, 2 cm.

Because of the bicontinuous phase coordination mechanism, our VHG materials have the capacity for programmable shape memory morphing. In this system, the PCL vitrimer, acting as a penetrated framework phase, dominantly provides the shape memory effect. Simultaneously, the ILgel intensifies the VHG network reconfiguration with its stable interfacial catalytic effect. Figure 4A exhibits the Nano CT scanning, capturing the VHG_5_’s bicontinuous structure variations during the programmable shape memory process. Above its *T*_m_, the softened VHG_5_ was programmed to a temporary shape via stretching. When cooled below *T*_m_, VFP crystallized, solidifying the VHG’s temporary shape. Reheating above *T*_m_ allowed the VHG material to recover to its original shape state. By activating the bicontinuous network reconfiguration, this temporary shape could also be memorized and programmed into a permanent one. The Nano CT scans demonstrated a consistent biphasic structure between the temporary and permanent shape states. We also quantified the shape fixity ratio (*R*_f_), shape recovery ratio (*R*_r_), and shape memorization ratio (*R*_m_) of different VHGs (fig. S16). *R*_f_, *R*_r_, and *R*_m_ of VHG_7.5_ were close to 100%, and those of VHG_2.5_ exceeded 97%. As shown in Fig. 4B, highly programmable shape memory morphings of VHG_5_ were based on the Miura origami and square-patterned kirigami. In addition, in combination with its high-strain performance, VHG_5_ exhibited fully recoverable strains of up to 1000% through thermal stimuli, and its recovery response time was less than 1 s, indicating its ultrafast shape memory recovery properties (movie S1).

### Bidirectional stiffness-gated iontronic piezoresistivity

Piezoresistive iontronic sensors, which convert external mechanical effects into recognizable ion-to-electron signals, have found wide applications in smart electronics and actuators (*25*–*27*). Currently, the piezoresistive effect in ion hydrogels and ILgels is primarily based on ion transmission and redistribution under mechanical stress, resulting in corresponding changes in materials’ conductivity and resistance (*28*). In existing gel iontronics with homogeneous network structures, the negative piezoresistivity can be observed, where resistance decreases under compressive strain. In contrast to the traditional negative piezoresistivity, the positive piezoresistive sensors have also been developed (*29*, *30*), which have exhibited the increased compressive resistance and the corresponding decrease in conductivity. For example, by introducing liquid metal into the magnetorheological elastomer, the heterogeneous composites exhibited an unconventional positive piezoconductive effect (*30*). However, whether the piezoresistive property is positive or negative, current sensor systems show unidirectional intrinsic features. Such unidirectional sensing restricts the sensor’s ability to achieve sophisticated awareness to some extent. To date, iontronic sensor systems that integrate both positive and negative piezoresistive properties, along with high-sensitivity and high-strain capabilities, have remained underexplored.

**Fig. 5.**
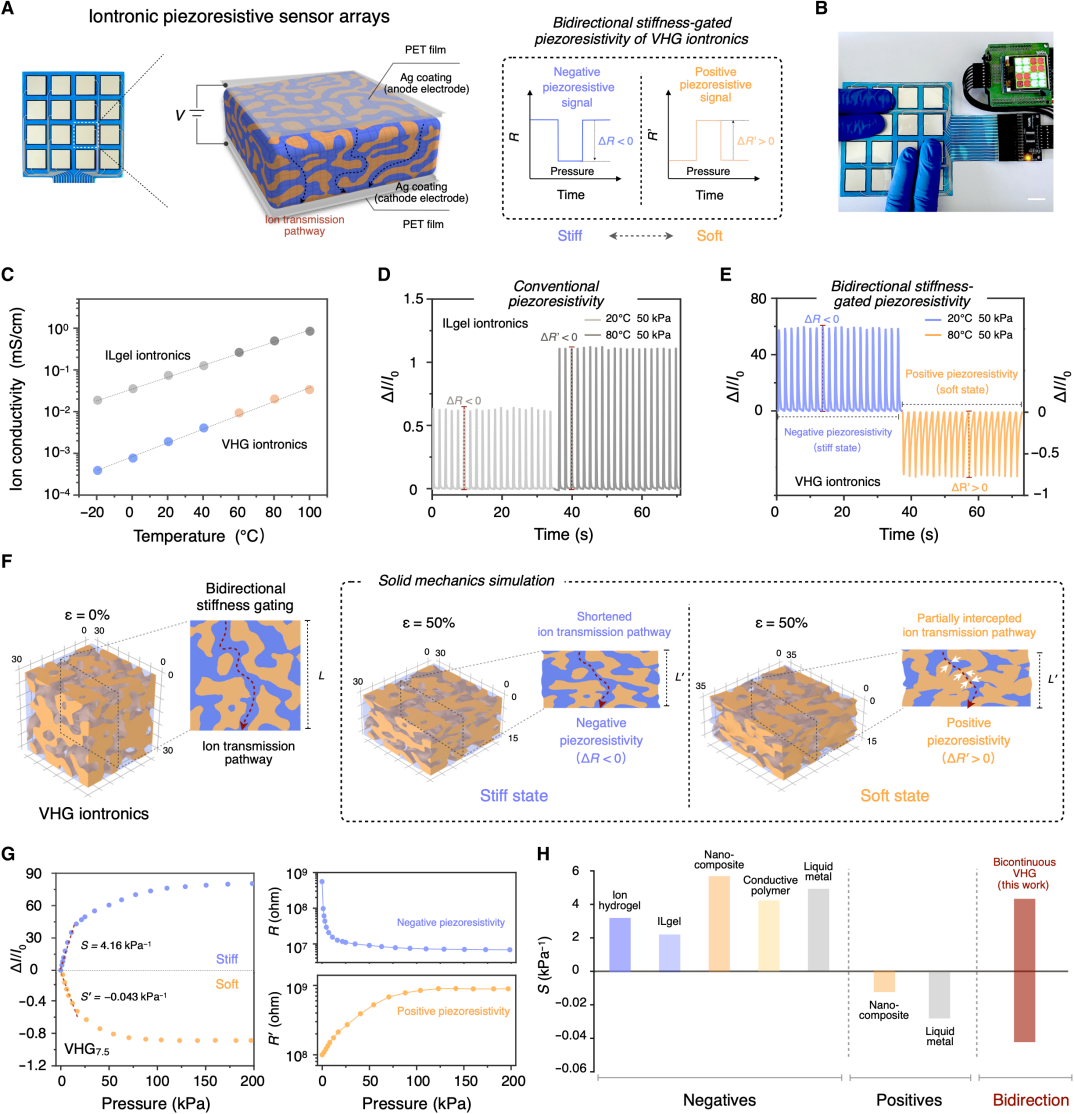
Bidirectional stiffness-gated iontronic piezoresistivity. (**A**) Schematic illustration of VHG iontronic piezoresistive sensor (4 × 4) arrays that had the capability of the bidirectional stiffness-gated piezoresistivity. Scale bar, 1 cm. (**B**) When the VHG iontronic sensor units were pressed, the corresponding feedback was reflected on the monitor. (**C**) Ionic conductivity of the ILgel iontronics and VHG_7.5_ iontronics ranged from −20° to 100°C. (**D**) Negative piezoresistive signal (Δ*I*/*I*_o_) responses of the conventional ILgel iontronics under loading (loading pressure, 50 kPa) and unloading cycles, respectively. (**E**) Distinct negative and positive piezoresistive signal responses of the VHG_7.5_ iontronics. (**F**) Three-dimensional finite element analysis of VHG solid mechanics, demonstrating the variations of the ion transport pathway during the bidirectional stiffness-gated piezoresistivity process. (**G**) Negative and positive piezoresistive signal responses of the VHG_7.5_ iontronics against different pressures, respectively. (**H**) Distinct from the unidirectional negative/positive features of any existing piezoresistive systems, VHG iontronic systems exhibited a bidirectional stiffness-gated piezoresistivity.

In our systems, VHGs have a switchable stiffness-gating ion transmission pathway that was closely correlated with their two primary tunable mechanical states: stiff and soft (Fig. 5A). Therefore, the bidirectional stiffness-gated positive and negative piezoresistivity of VHG iontronics can be realized. The piezoresistive sensor (4 × 4) arrays were constructed by encapsulating the VHG film between two poly (ethylene terephthalate) films coated with Ag electrodes. When the VHG iontronics sensor units were pressed, the corresponding feedback was vividly displayed on the monitor (Fig. 5B). To further investigate such bidirectional stiffness-gated piezoresistive effects, we first confirmed the ionic conductance of both ILgel iontronics and VHG iontronics. Figure 5C shows that the ion conductivity of VHG was lower than the ILgel, which can be related to the ion shielding effect from the VFP structure. As the temperature increased over a wide range from −20° to 100°C, the trends in ion conductivity variation remained consistent, which can be attributed to the positive and linear correlation of their ionic transfer efficiency with temperature. As a result, during loading (with a loading pressure of 50 kPa) and unloading cycles, the piezoresistive signal response of the ILgel iontronics demonstrated some sensitivity differences at 20° and 80°C. In both cases, the corresponding Δ*I*/*I*_o_ values were positive, indicating a typical unidirectional negative piezoresistive signal (Δ*R* < 0) response (Fig. 5D). In contrast, under the same mechanical field, the Δ*I*/*I*_o_ response of the VHG iontronics integrated distinct positive and negative signals (Fig. 5E). In the stiff state, the VHG iontronic sensor showed the negative piezoresistivity sensing behavior (Δ*R* < 0) that was similar to that of the ILgel iontronics. The VHGs’ high sensitivity can be attributed to the bicontinuous structure of the soft IFP and the stiff VFP, which constructs a continuous ionic transport pathway that allows for more efficient ion transmission and redistribution under mechanical stress. When transitioning to a soft state at 80°C, the VHG iontronics demonstrated a shift to positive piezoresistivity (Δ*R*′ > 0). These results indicate that VHG iontronics can self-adapt in response to external stimuli, achieving bidirectional piezoresistivity with high-sensing properties owing to the mechanical ion-gating properties of the bicontinuous phase structure.

To further elucidate such bidirectional stiffness-gated piezoresistive mechanism, we conducted a solid mechanics simulation associated with variations in VHG systems’ ionic transmission pathways through a three-dimensional finite element analysis (Fig. 5F). In the simulation setup, *E*_stiff_ and *E*_soft_ of VFP were set to 208 MPa and 74 kPa, respectively, and those of the IFP were set to 1.8 and 1 kPa, respectively. These values were chosen on the basis of related actual mechanical results. We set the overall shape deformation of the bicontinuous structures at 50%. In the stiff VHG state, the presence of the VFP and the IFP heteronetwork provided a contrasting mechanical feature, and their related Poisson ratios were 0.1 and 0.3, respectively. When VFP, acting as an ion shielding phase, was compressed axially, the deformation of the stiff vitrimer resembled folding, leading to limited lateral expansion. The shortened ion transmission pathway within the IFP induced a more pronounced change in ionic conductivity under applied stress, demonstrating the negative piezoresistivity behavior of the stiff VHG. In the case of the softened VHG state, the PCL vitrimer phase became flexible and was easily deformed under compression, which affected the deformation experienced by the soft ILgel phase. Thus, the softening deformation partially intercepted the continuous ion-conducting pathways, further leading to positive piezoresistivity.

We then explored the bidirectional stiffness-gated piezoresistivity properties in different VHG systems (Fig. 5G and fig. S17). The sensitivity (*S*) of the iontronic piezoresistive sensors was defined as *S* = (Δ*I*/*I*_o_)/*P*, in which *P* represents the applied pressure. By combining the bicontinuous structure with wide-span switchable stiffness and iontronic coordination, we found that all VHG iontronics with different VFP and IFP components exhibited bidirectional piezoresistive sensing properties with diverse sensitivity over a large pressure range of 0 to 200 kPa. The stiff VFP of VHG_7.5_ provided high mechanical support and stable mechanotransduction, indicating a high sensitivity (*S* = 4.16 kPa^−1^) of negative piezoresistive signals. Conversely, the deformation of the softened VFP resulted in the disruption of ion pathways and decreased ion mobility, resulting in notable positive piezoresistivity upon compression. The ILgel iontronics only exhibited a negative piezoresistivity with relatively low sensitivity (*S* = 0.15 kPa^−1^; fig. S18). Moreover, the remarkable shape reconfiguration capabilities of VHGs allowed for constructing microstructures, such as micropillars and microribbons on their surfaces, improving their sensing performance diversity (fig. S19). Figure 5H and table S5 present a comparison between the VHG iontronics and existing unidirectional piezoresistive systems, including ion hydrogel, ILgel, nanocomposite, conductive polymer, and liquid metal. VHG iontronic sensor systems demonstrated a unique bidirectional stiffness-gated piezoresistivity that integrated both positive and negative piezoresistive properties, combined with diverse sensitivity and shape reconfiguration, effectively elevating the sensor’s sophisticated awareness capabilities.

## DISCUSSION

In this work, we used a phase-engineering strategy to develop a vitrimer heterogeneous gel material. Such VHGs achieved wide-span switchable stiffness and iontronic coordination, where the bicontinuous structure offered a crucial synergistic architecture to optimize materials’ programmable properties. Owing to the synergistic effects of the bicontinuous phases and interfaces, VHGs had high mechanical stiffness with an elastic modulus of up to 116 MPa, and a high-strain performance of more than 1000%. The maximal ratio of switchable stiffness in VHG systems surpassed 5 × 10^3^. Owing to the ion transmission pathway that responds dynamically to mechanical stimuli, our VHG iontronics construct a unique bidirectional stiffness-gated piezoresistivity, integrating both positive and negative piezoresistive properties. Such bidirectional piezoresistivity features are different from any current unidirectional piezoresistive sensing system, which can greatly enhance the sensor’s sophisticated awareness capabilities. Otherwise, specific ion liquids act as the ILgel dispersion phase and stable interfacial accelerators, improving the VHG network reconfiguration for highly programmable reprocessing and shape memory effect. Owing to these functionalities, we expect that VHGs will offer an important material foundation for possible smart devices to suit a variety of complex applications. Moreover, our bicontinuous design concept has then potential for expansion to other gel or vitrimer systems with functionalities like smart adhesion or mechanical gating transmission, which will offer a versatile platform for smart sensors, soft machines, and bioelectronics.

## MATERIALS AND METHODS

### Materials

PCL-diol [number-average molecular weight (*M*_n_) ~10,000] was purchased from Sigma-Aldrich. THDI (Desmodur N 3900) was purchased from Covestro. HFBA was purchased from Aladdin. DBTDL, ethyleneglycol dimethacrylate, toluene, and phenylbis (2,4,6-trimethylbenzoyl) phosphine oxide (PBPO; photoinitiator) were purchased from J&K Chemical Ltd., China. A series of ionic liquids (ILs) with different cation and anion structures were purchased from Lanzhou Yulu Fine Chemical Co. Ltd. (GanSu, China). All available chemicals were used without any further purification unless otherwise noted.

### Fabrication of the bicontinuous VHGs

The bicontinuous VHGs were fabricated using an orthogonal polymerization-induced phase separation strategy. For example, 1 g of PCL-diol, 0.5 g of HFBA, 0.5 g of IL, and PBPO (0.1 wt % to HFBA) were combined and stirred to obtain a homogeneous solution. Then, 0.03 g of THDI was quickly added. The VHG_5_ materials were fabricated via the orthogonal polymerization reaction under UV light (405 nm) and thermal conditions (80°C) for 4 hours. Subsequently, the cured VHG_5_ were placed at 80°C for 24 hours. The samples were denoted as VHG_−*x*_, where *x* represents the weight ratio of VFP to VHG (that was fixed to 10).

### Fabrication of the PCL vitrimers

The PCL vitrimers were synthesized using a polyurethane reaction. Two grams of PCL-diol, 0.3 g of toluene, and 0.06 g of THDI were combined and stirred for several minutes. The mixture was poured into a mold, and curing was conducted thermally at 80°C for 24 hours. Last, the cured sample was placed at 80°C for 24 hours.

### Fabrication of the ILgels

One gram of HFBA, 1 g of IL, and PBPO (0.1 wt % to HFBA) were mixed and stirred to generate a stable ILgel precursor. Next, ILgels were prepared using the UV (405 nm) free-radical polymerization for 4 hours at room temperature.

### Fabrication of the IL-PCL vitrimers

The IL-PCL vitrimers were synthesized using a polyurethane reaction. Two grams of PCL-diol, 0.06 g of THDI, and 0.5 g of IL were combined and stirred for several minutes. The mixture was poured into a mold, and curing was conducted thermally at 80°C for 24 hours. Last, the sample was placed at 80°C for 24 hours.

## Supplementary Materials

### This PDF file includes:

Supplementary Text Figs. S1 to S19 Tables S1 to S5 References
