# Supplementary material for: Bicontinuous vitrimer heterogels with wide-span switchable stiffness-gated iontronic coordination
Source: Sci Adv. 2024 Mar 8;10(10):eadl2737. doi: 10.1126/sciadv.adl2737 (PMC10923496; doi:10.1126/sciadv.adl2737)
Supplement: Supplementary file 1 — Supplementary Text Figs. S1 to S19 Tables S1 to S5 References [file sciadv.adl2737_sm.pdf]

Science Advances
AAAS

Supplementary Materials for

Bicontinuous vitrimer heterogels with wide-span switchable stiffness-gated iontronic coordination

Ziguang Zhao *et al.*

Corresponding author: Ziguang Zhao, zhaoziguang@ucas.ac.cn; Yuchen Wu, wuyuchen@iccas.ac.cn; Mingjie Liu, liumj@buaa.edu.cn

Sci. Adv. **10**, eadl2737 (2024)
DOI: 10.1126/sciadv.adl2737

The PDF file includes:

Supplementary Text
Figs. S1 to S19
Tables S1 to S5
References

Other Supplementary Material for this manuscript includes the following:

Movie S1

Supplementary Text:

AFM characterization

The MultiMode 8 atomic force microscope (AFM) with NanoScope V controller from Bruker (Bruker Corporation, Santa Barbara, CA) equipped with PeakForce Quantitative Nanomechanical Mapping (PF-QNM) mode was used for samples topography imaging and nanomechanical mapping under ambient conditions. Previously, the VHG precursor was cast on a silicon wafer and then cured, preparing a VHG sample with a flat surface. NPS (Bruker′s antimony (n) doped silicon probes with a nominal spring constant of 40 N m^{-1} and a tip radius of 10 nm) type probes were chosen. In PF-QNM mode AFM, the deflection sensitivity and the spring constant for each cantilever were calibrated using the built-in cantilever calibration, the ramp, and thermal noise method, respectively. All AFM experiments were performed under ambient conditions. An AFM offline processing system, the NanoScope Analysis software, was used for morphology and surface modulus value measurements and analysis.

Nano CT characterization

A high-resolution Nano CT device (Bruker SkyScan2214CMOS, Germany) was used to scan the specimens at a voxel size of 100 nm, 60 kV, 100μA, 360 ° of rotation, a 0.08 ° of rotation step, 4 of averaging frames. The VHG samples were cut to the specific size (800 μm height × 800 μm width × 1 mm thickness). Rotating 360 degrees for data collection yielded a total of 4500 projection images. The data were then imported into NRecon software for reconstruction, resulting in a total of 2968 slice data. The data volume represented a cylinder with a diameter of 500 μm and a height of approximately 290 μm. Subsequently, a 100 μm side length cube was selected from the data. After applying filtering in the CTAn software, the image results were subjected to threshold segmentation. The data representing the two-phase structures were concurrently imported into CTVox, where color rendering was performed, yielding the image results.

SEM and FTIR characterization

Scanning electron microscopy (SEM) image of the PCL vitrimer framework was recorded on a SEM (HITACHI S-4800). The sample was prepared by utilizing the solvation effect of ethanol, undergoing three cycles of soaking for 12 hours and washing with ethanol to remove the ILgel phase of the VHG_5. The sample was sputtered with a layer of Au using the magnetron sputtering instrument for SEM imaging. Moreover, the PCL vitrimer was prepared and used to characterize the infrared spectra by a Fourier-transform infrared (FTIR) spectrometer (Vertex 70, Bruker).

Mechanical measurements

The tensile and compressive tests were conducted using a tensile-compressive tester (Mark-10/ESM301). In the tests, dog bone-shaped samples (15 mm length × 5 mm width × 1 mm height) were subjected to in the tensile tests at a rate of 50 mm min^{-1}. The tensile strain (ε) was determined as the elongation (ΔL) divided by the initial length (L_0) ($\varepsilon = \Delta L / L_0 \times 100\%$). The elastic modulus ($E$) was calculated from the initial linear region of stress-strain curve. Cylinder-shaped samples (5 mm diameter × 10 mm height) were used for compressive tests with a deformation rate of 10% of the sample height per minute.

Thermal analysis

A TGA analyzer, the Perkin-Elmer TGA 4000, was used to perform the thermogravimetric analysis (TGA) measurements. The VHG was heated at a rate of 5 °C min^{-1} with a dry nitrogen stream from room temperature to 500 °C. Differential scanning calorimetry (DSC) data were obtained using a TA Instrument (DSC Q2000) under dry nitrogen environment. The samples, sealed in aluminum pans, were scanned between 10 and 80°C at a scanning rate of 5 °C min^{-1}.

Rheological test

The rheological properties of the samples were investigated by a modular compact rheometer (Anton Paar, MCR 301). For frequency sweeps, a 15 mm parallel plate geometry was used with a 1 mm gap size at a constant temperature of 80 °C and strain of 0.1%. For the gelation time

measurement, a 30 mm parallel plate was used instead to improve the signal quality, as the samples were liquid-like. Measurements were taken at 80 °C, 15.8 rad/s, and a strain rate of 0.1%. Under a variety of temperatures, the storage modulus (G') of the samples was swept in a range of 0.1 ~ 100 rad s^{-1} or at 15.8 rad s^{-1} at a constant strain of 0.1 %. For creep measurements of the VHG_5, a shear stress (10 Pa) was first applied for up to 45 min. Subsequently, the stress was removed, and the sample was allowed to recover for 45 min.

Stress relaxation

Stress relaxation tests were carried out using dynamic mechanical analysis (DMA Q800, TA instruments) in a "stress relaxation" mode with a strain value set at 10%. In stress relaxation tests, the samples were stretched under a constant strain of 10% in the temperatures ranging from 100 to 140 °C. The heating rate was 5 °C min^{-1}.

Swelling-deswelling tests

Weighted PCL-vitrimers (0.5 g) were soaked in 10 g of toluene for 24 h at room temperature until they reached reaching the swelling equilibrium. The samples were subsequently dried under vacuum for 24 h at 80 °C until reaching a constant value. During five cycles, the vitrimer contents were calculated as: 100% × final weight/ initial weight.

Shape memory and shape reconfiguration test

Shape fixity ratio (R_f) and shape recovery ratio (R_r) were computed as indicators of the shape memory property. The equations to calculate the parameters R_f and R_r are as follows:

$$R_f = \varepsilon_f / \varepsilon \times 100\%,$$

$$R_r = (\varepsilon_f - \varepsilon_r) / \varepsilon_f \times 100\%,$$

where ε, ε_f, and ε_r represent the strain under deformation at a temperature above T_m, the fixed strain in the temporary shape, and the original shape′s strain after shape recovering process, respectively.

The equations to calculate the parameters R_m and R_{re} are as follows:

$$R_m \ or \ R_{re} = \varepsilon_p' / \varepsilon_p \times 100\%,$$

where ε_p and ε_p' represent the processed strain induced by activating the bicontinuous network reconfiguration at 130 °C for 4 hours under an applied external force, and the sustained strain by reheating above T_m.

Piezoresistivity measurement

The piezoresistivity performance of the piezoresistive sensors was evaluated on a homemade test system consisting of a forcemeter (Mark-10/ESM301) and electrochemical workstation (CH Instruments, CHI660E). We tested the ion conductivity of both ILgel-iontronics and VHG-iontronics with the temperature from -20 to 100 °C through the amperometric i-t test model. The piezoresistive sensor (4 × 4) arrays were constructed by encapsulating the VHG film between two poly (ethylene terephthalate) films coated with Ag electrodes.

Finite element modeling

Finite element modeling (COMSOL Multiphysics6.0, MA, USA) was employed to simulate the ionic transport pathway of the bicontinuous phase structure under deformation, the 3D model was built from 3DS MAX 2020(Autodesk, USA). In the simulation setup, Young's modulus of VFP at room temperature and high temperature were set as 208 MPa and 74 kPa, and Poisson ratios were 0.1 and 0.3 with the same density which was 1.1 g/ml. Young's modulus of IFP was 1.8 kPa at both room temperature and high temperature, that Poisson ratio was 0.3, and the density was 1.46 g/ml. The overall shape variable of the structure is set to 50%.

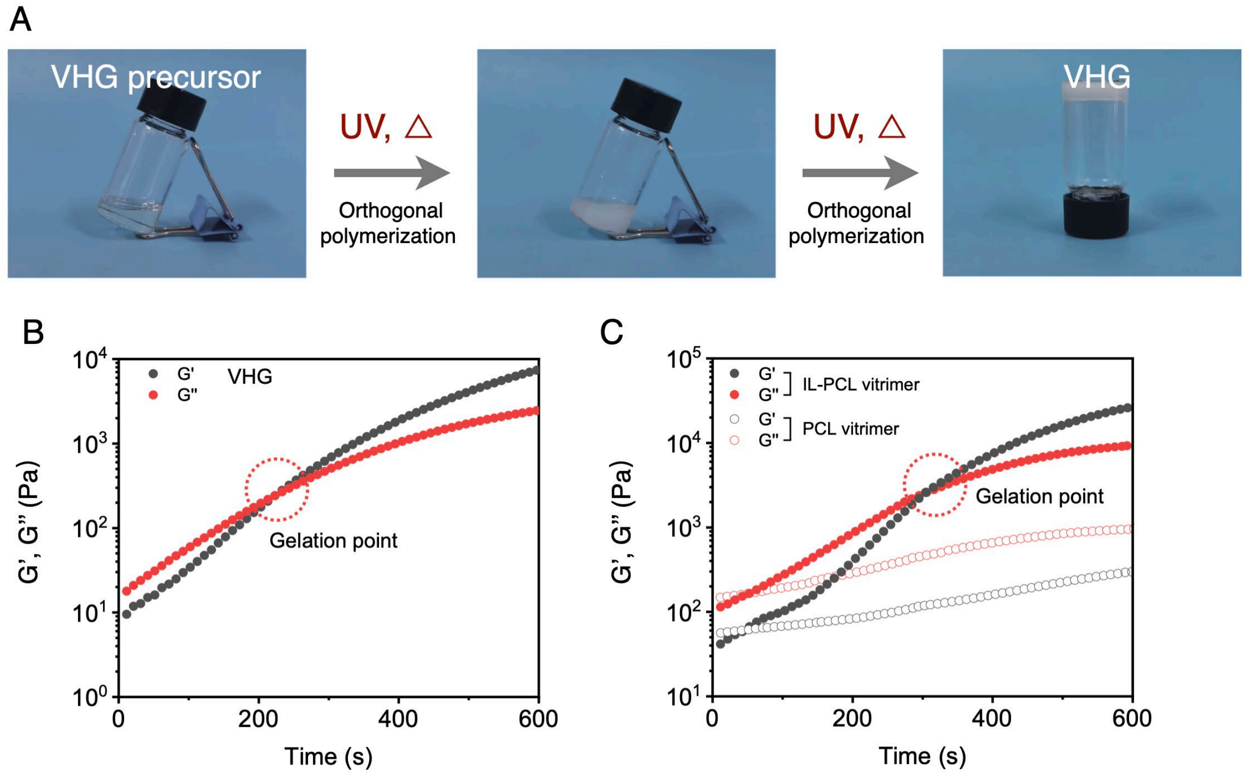

Fig. S1. The gelation process of the VHG. (A)The "one-step"orthogonal polymerization of the VHG material. (B) The gelation point of the VHG material. (C) The storage modulus (G') and loss modulus (G'') of the catalyst-free PCL vitrimer and the IL-PCL vitrimer during the gelation process.

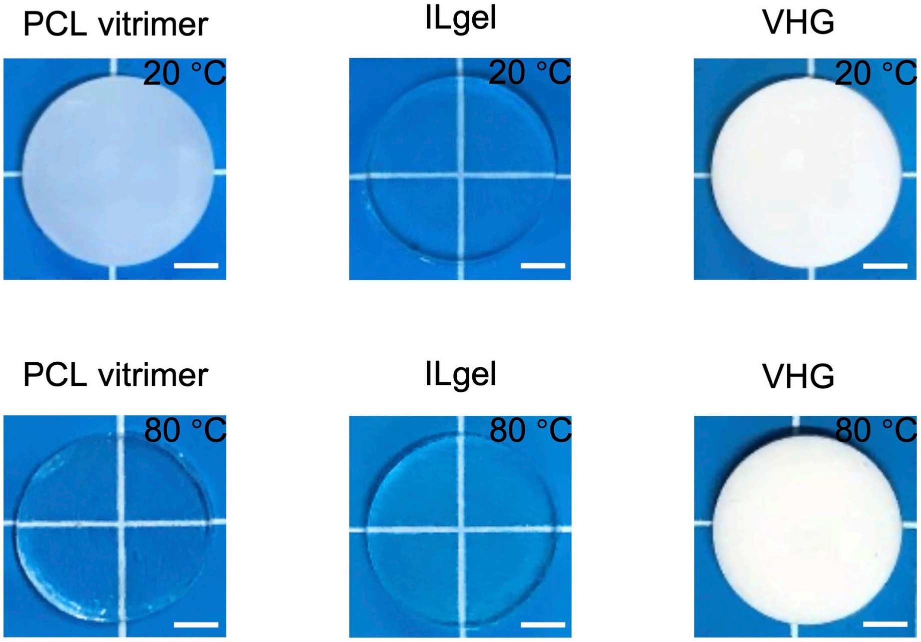

Fig. S2. Optical photographs of the PCL vitrimer, the ILgel, and the VHG at 20 °C and 80 °C, respectively. Scale bar, 0.5 cm.

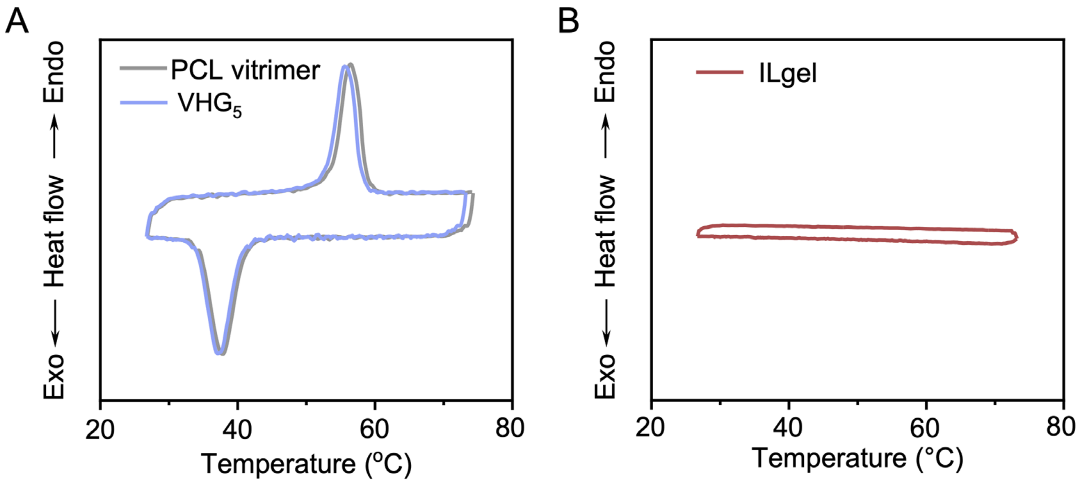

Fig. S3. DSC curves of the PCL vitrimer and the VHG_5 (A) and the ILgel (B).

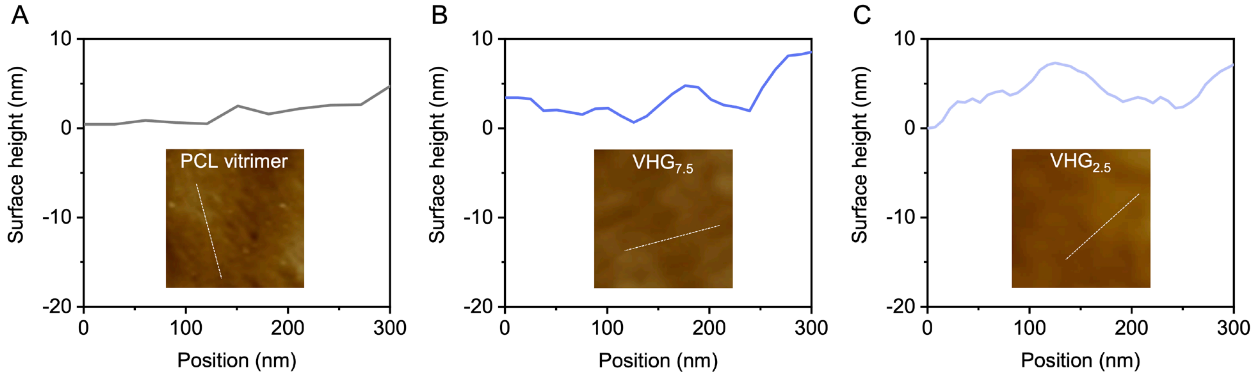

Fig. S4. Surface height variation of the PCL vitrimer (A), the $VHG_{7.5}$ (B), and the $VHG_{2.5}$ (C).

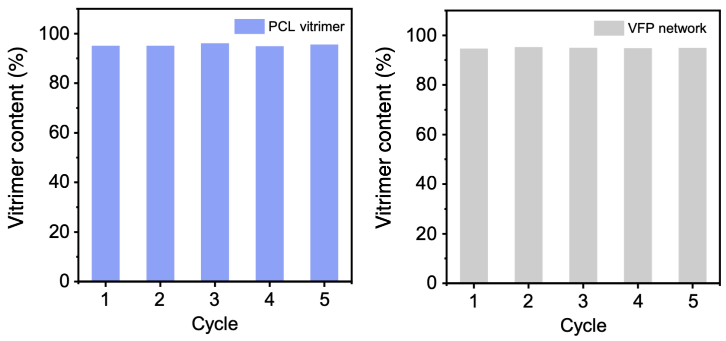

Fig. S5. The contents of the VFP network and the PCL vitrimer during the swelling-deswelling cycles.

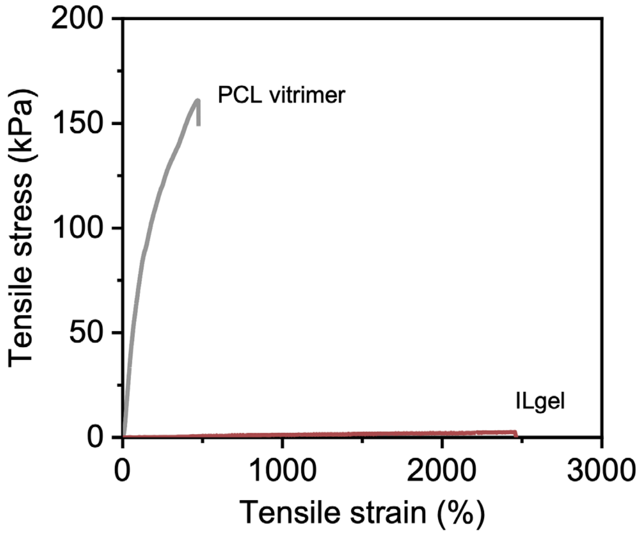

Fig. S6. Tensile stress-strain curves of the PCL vitrimer and the ILgel at 80°C.

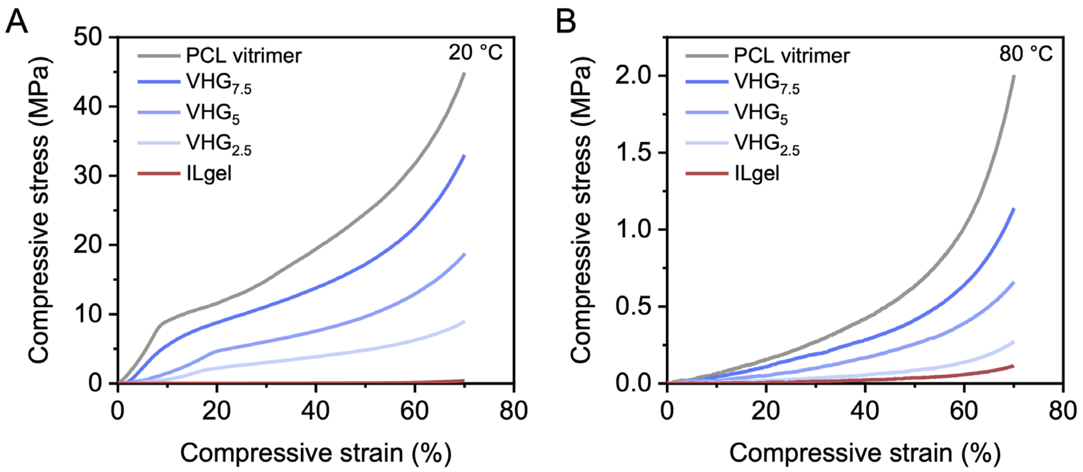

Fig. S7. Compressive stress-strain curves of the PCL vitrimer, the ILgel, and the VHGs with different VFP and IFP components at 20 °C (A) and 80 °C (B), respectively.

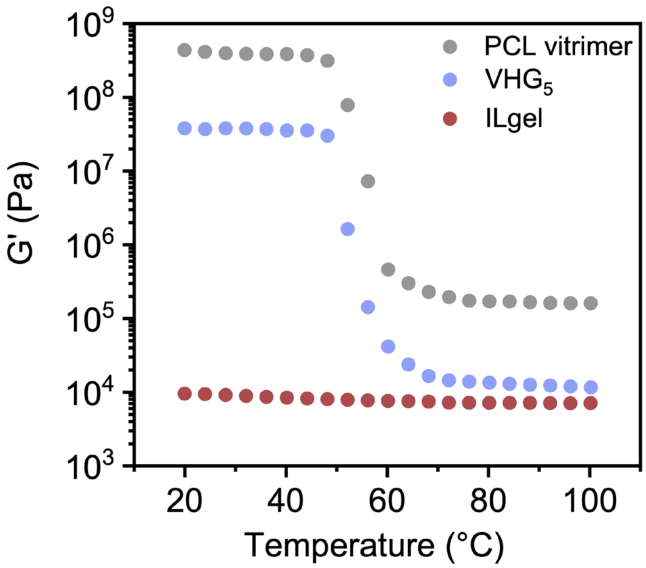

Fig. S8. Storage moduli (G') of the PCL vitrimer, the VHG_5, and the ILgel on a temperature sweep in the range of 20 to 100 °C.

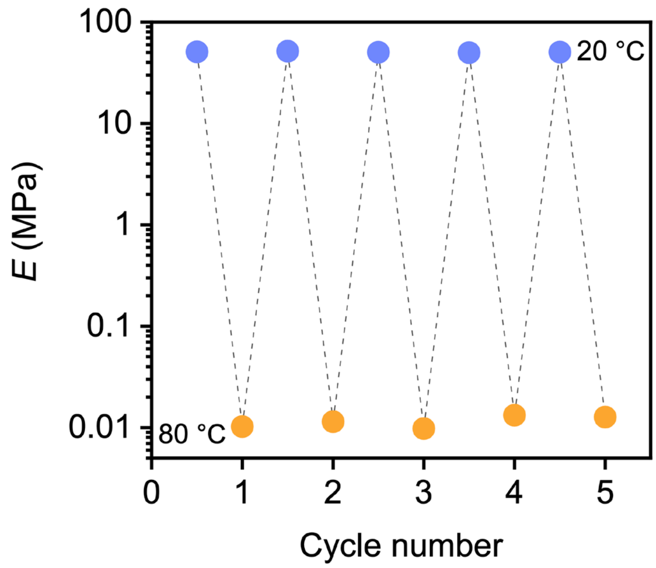

Fig. S9. Stable transitions between high and low elastic modulus of the VHG_5 at 20°C and 80 °C.

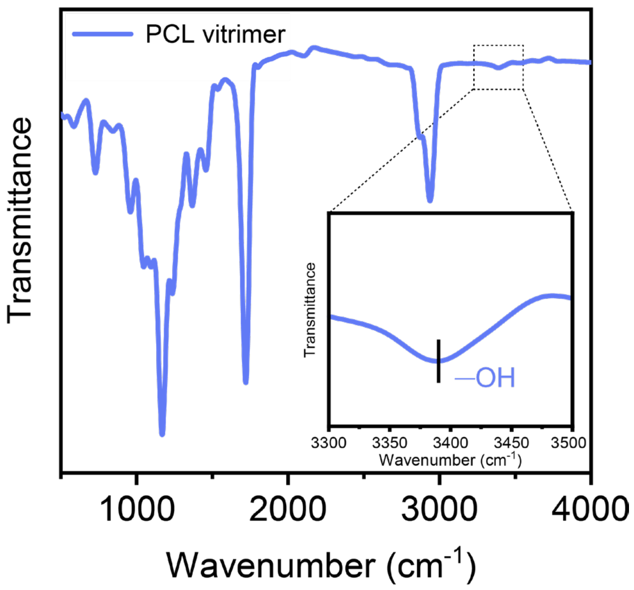

Fig. S10. The FTIR spectrum exhibited the characteristic absorption peaks of the residual hydroxyl groups within the PCL vitrimer.

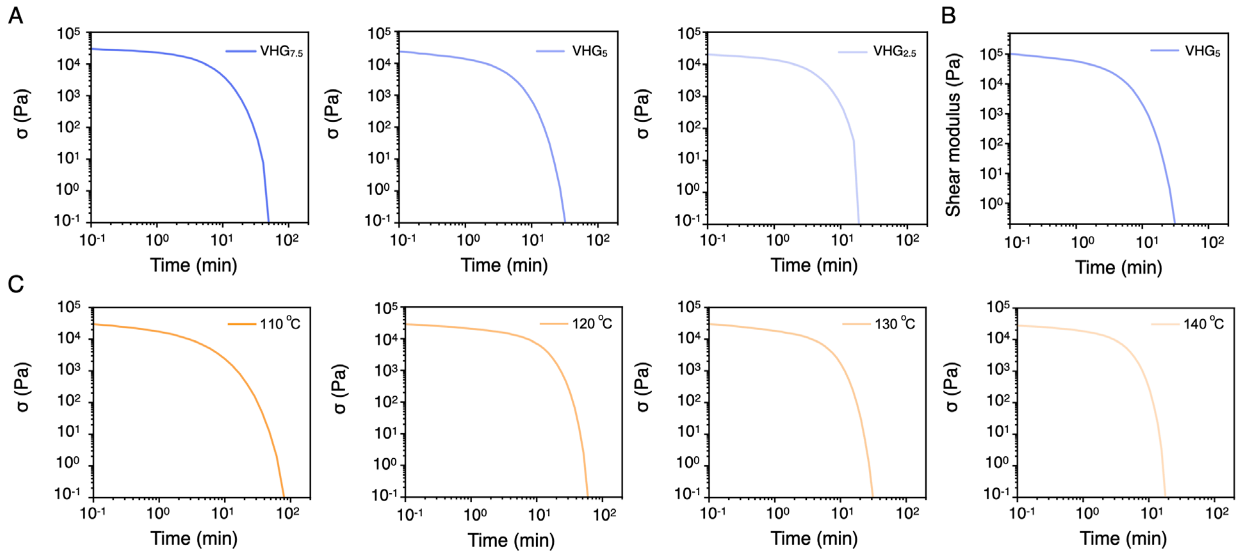

Fig. S11. Stress relaxation tests. (A) Stress relaxation behaviors of the VHGs with different VFP and IFP components at 130 °C. (B) The related shear modulus of the VHG_5. (C) Stress relaxation behaviors of the VHG_5 as the temperature increased from 110 °C to 140 °C.

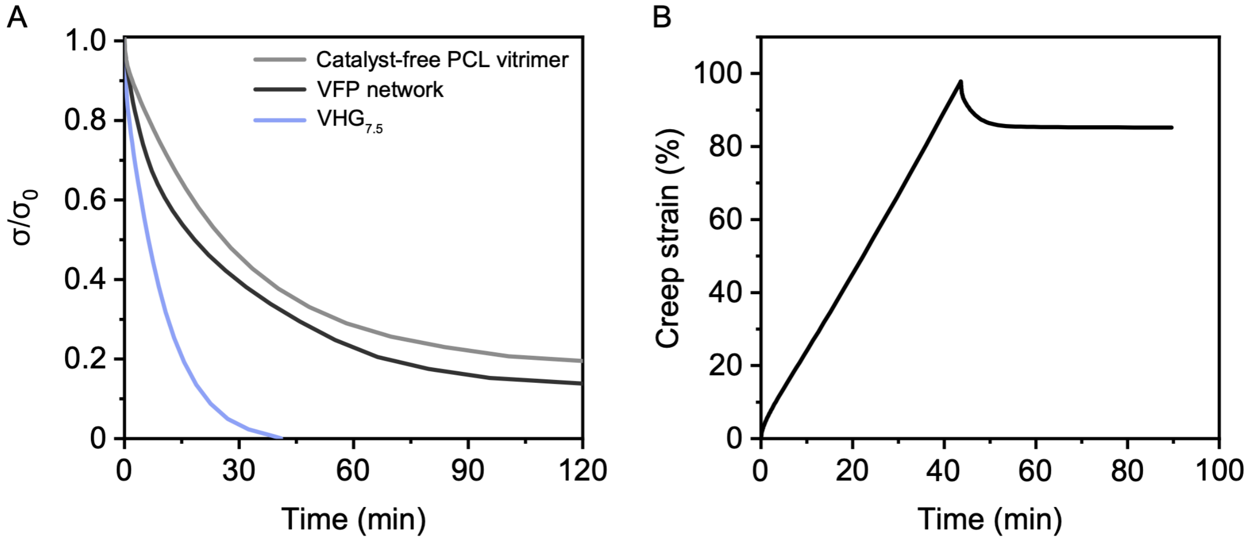

Fig. S12. Stress relaxation behaviors of the catalyst-free PCL vitrimer, the VFP network, and the $VHG_{7.5}$ at 130 °C (A), and the creep curve of the VHG_5 at 130 °C under applied shear stress of 10 Pa (B).

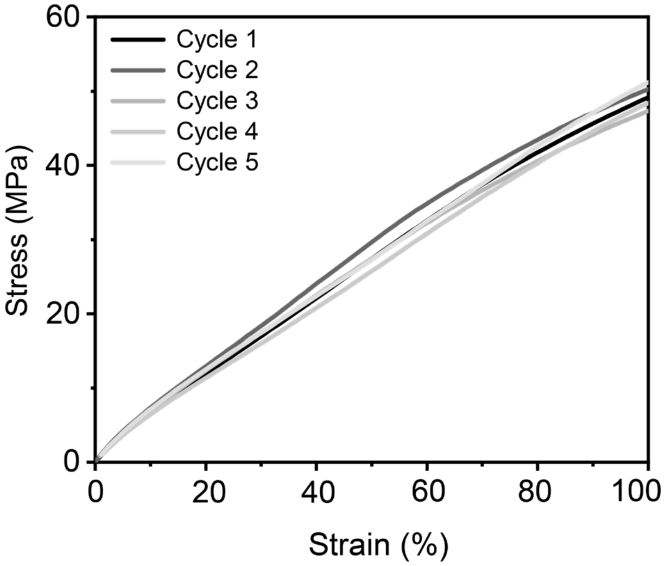

Fig. S13. Stable mechanical performance of the VHG_5 across multiple reprocessing cycles.

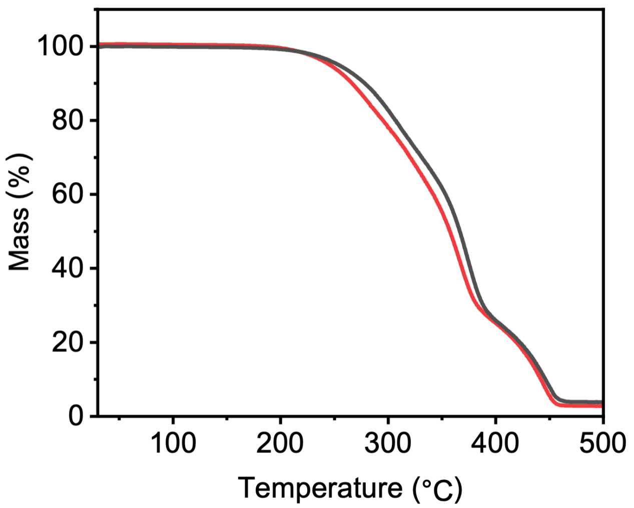

Fig. S14. TGA analysis of the original VHG (black line) and the VHG samples after stress relaxation (red line).

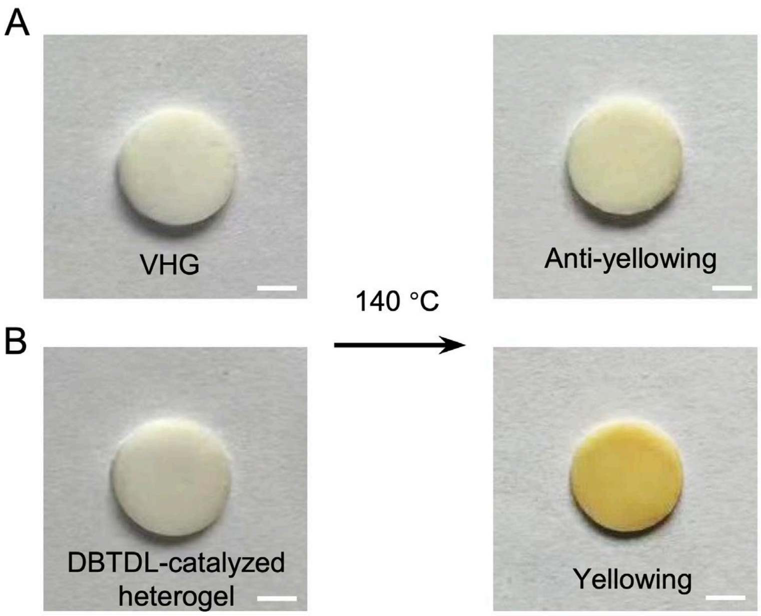

Fig. S15. Optical photographs of VHGs displayed remarkable anti-yellowing effect compared to the DBTDL-catalyzed heterogel system. Scale bar, 0.5 cm.

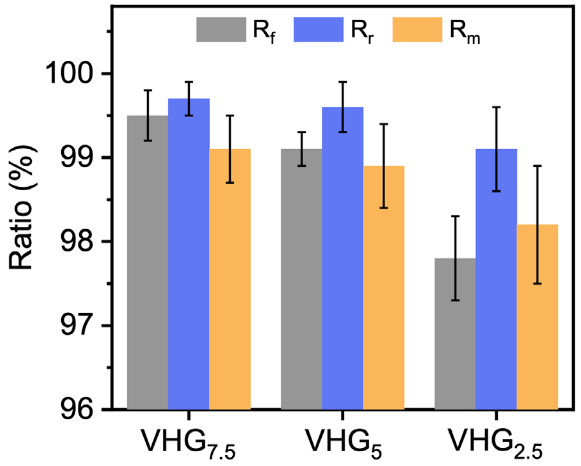

Fig. S16. Shape fixity ratio (R_f), shape recovery ratio (R_r), and shape memorization ratio (R_m) of the VHGs with different VFP and IFP components.

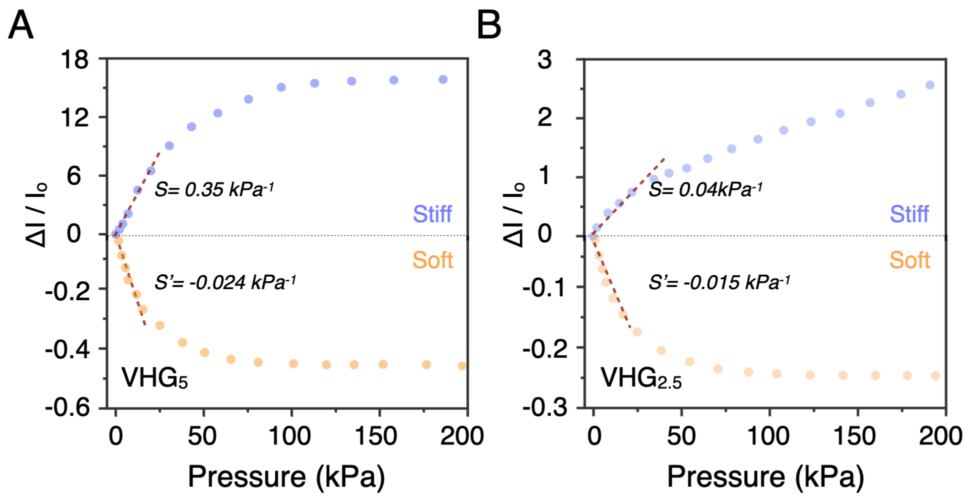

Fig. S17. Negative and positive piezoresistive signal responses of the VHG_5 iontronics (A) and $VHG_{2.5}$ iontronics (B) against different pressures, respectively.

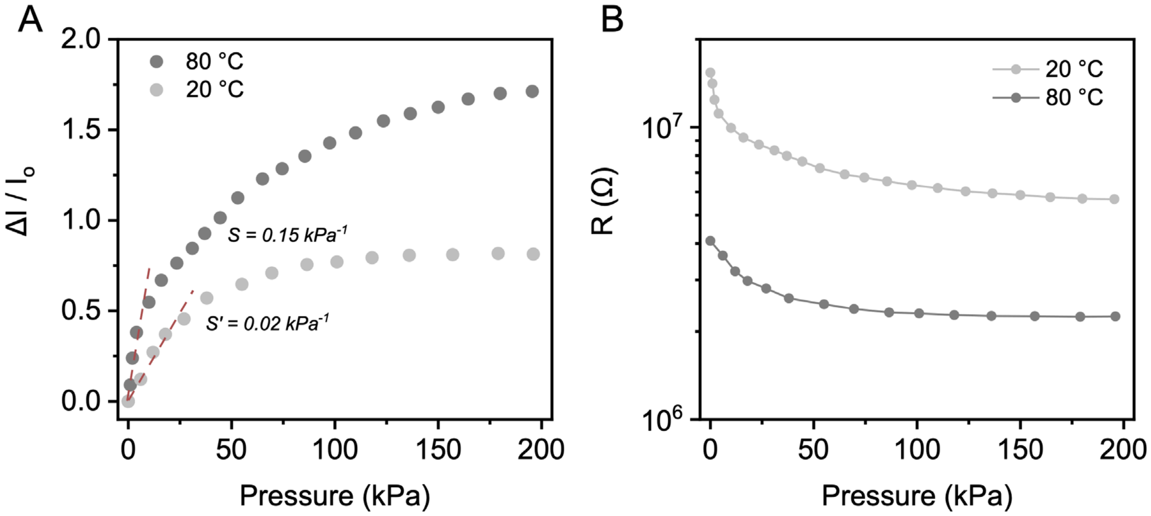

Fig. S18. Negative piezoresistive signal (A) and resistance (B) responses of the ILgel-iontronics against different pressures at 20 °C and 80 °C, respectively.

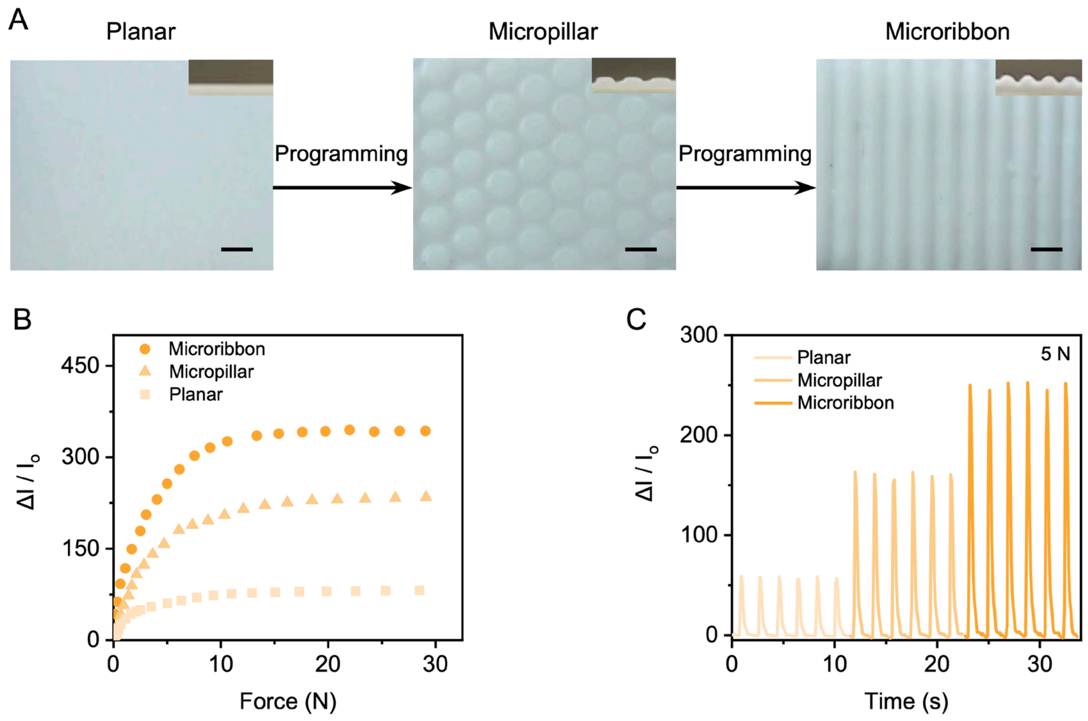

Fig. S19. Sensing performance diversity of the VHG. (A) Optical photographs of the $VHG_{7.5}$ with different microstructures (planar, micropillar, and microribbon). (B) Negative piezoresistive signal responses of the $VHG_{7.5}$ iontronics with different microstructures and sensitivity to various forces at 20 °C. (C) Negative piezoresistive signal responses of the $VHG_{7.5}$ iontronics with different microstructures under loading/unloading cycles at 20 °C. Scale bar, 1 mm.

Table S1. Comparison of the switchable stiffness ratio and strain$_{max}$ for VHGs with previously reported typical switchable mechanics polymer materials, including vitrimers, shape memory polymers and gels, organohydrogels, stimuli-responsive gels, and ILgels.

	Switchable stiffness ratio	Strain$_{max}$ (%)	Reference
Shape memory polymer	146	78	31
	1000	930	32
	6500	300	33
	2.8	322	34
Shape memory gel	8.8	530	35
	165	280	36
	4.4	900	37
Stimuli-responsive hydrogel	663	650	38
	4.1	1800	39
	400	170	40
Organohydrogel	27.7	1500	20
	12.9	520	41
	23.7	1400	42
Ion-liquid gel (ILgel)	137742	560	7
	20	360	15
	1516.7	272	43
	2296.7	600	44
	1.2	255	45
	23142	850	46
	1723	1068	47
	2100	1100	48
	20	400	49
Vitrimer	16.7	800	2
	5000	400	12
	200	30	13
	2530	65	14
	667	210	50
	40000	50	51
	267	119	52
	1667	140	53
	1125	180	54
	1500	240	55
	55	150	56
	222	6	57
	210	13	58
	2000	110	59
**Vitrimer heterogel (VHG)**	**5100**	**2100**	**This work**

Table S2. Comparison of the elastic modulus (E_{stiff}) and strain$_{max}$ of VHGs with previously reported typical ILgels with different structures (i.e., single-/double-network, nanocomposite, phase-separated network, bicontinuous network).

	E_{stiff} (MPa)	Strain$_{max}$ (%)	Reference
Bicontinuous ILgel	85.4	560	7
	46.5	1200	60
Nanocomposite ILgel	16.4	686	61
	6.7	75	62
	5.6	12	63
	2.46	169	64
Microphase-separated ILgel	0.08	360	15
	0.13	720	65
	0.08	1100	66
	0.94	4500	67
	0.06	3000	68
Single-/ Double-network ILgel	55	5400	69
	5	450	70
	15.6	886	71
	82.8	1400	72
	0.5	2700	73
	0.197	2066	74
	63.1	5100	75
	0.484	5000	76
	0.067	1390	77
	0.05	1200	78
**Vitrimer heterogel (VHG)**	**116**	**2100**	**This work**

Table S3. Shape reconfiguration ratio of the VHGs fabricated by various ILs containing [NTf2] as the anion.

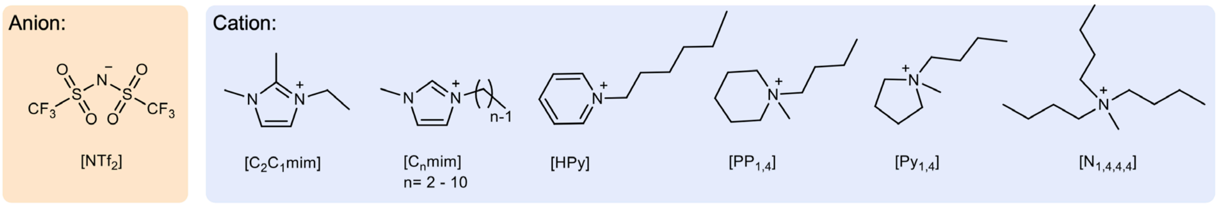

	[C_2C_1mim] [NTf_2]	[C_2mim] [NTf_2]	[C_{10}mim] [NTf_2]	[HPy] [NTf_2]	[$PP_{1,4}$] [NTf_2]	[$Py_{1,4}$] [NTf_2]	[$N_{1,4,4,4}$] [NTf_2]
Shape reconfiguration ratio (%)	98.5	98.1	97.8	97.3	97.8	97.6	97.6

Table S4. Various ion-liquids containing [$C_n$mim] as the cation were unable to produce a stable bicontinuous VHG structure.

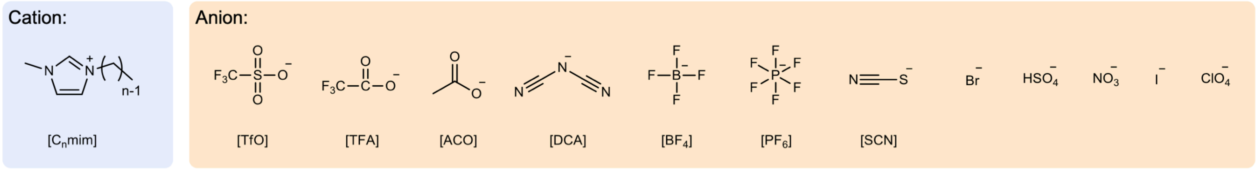

	[C_2mim] [TfO]	[C_2mim] [TFA]	[C_2mim] [ACO]	[C_2mim] [DCA]	[C_2mim] [BF_4]	[C_4mim] [PF_6]	[C_2mim] [SCN]	[C_6mim] [Br]	[C_2mim] [HSO_4]	[C_6mim] [NO_3]	[C_4mim] [I]	[C_2mim] [ClO_4]
Stable bicontinuous structure	No	No	No	No	No	No	No	No	No	No	No	No

Table S5. Distinct from the unidirectional negative/positive features of any existing piezoresistive systems, VHG-iontronic sensor systems exhibited a bidirectional stiffness-gated piezoresistivity.

	Positive sensitivity (kPa^{-1})	Negative sensitivity (kPa^{-1})	Reference
Ion hydrogel	2.83	/	79
ILgel	2.45	/	80
Nanocomposites	5.28	/	81
Conductive polymer	4.50	/	82
Liquid metal	4.31	/	83
Nanocomposites	/	-0.0054	84
Liquid metal	/	-0.025	85
**Bicontinuous VHG**	**4.16**	**-0.043**	**This work**

REFERENCES AND NOTES

1. D. Montarnal, M. Capelot, F. Tournilhac, L. Leibler, Silica-like malleable materials from permanent organic networks. *Science* **334**, 965–968 (2011).

2. M. Röttger, T. Domenech, R. Van Der Weegen, A. Breuillac, R. Nicolaÿ, L. Leibler, High-performance vitrimers from commodity thermoplastics through dioxaborolane metathesis. *Science* **356**, 62–65 (2017).

3. J. Deng, X. Kuang, R. Liu, W. Ding, A. Wang, Y. Lai, K. Dong, Z. Wen, Y. Wang, L. Wang, H. Qi, T. Zhang, Z. Wang, Vitrimer elastomer-based jigsaw puzzle-like healable triboelectric nanogenerator for self-powered wearable electronics. *Adv. Mater.* **30**, e1705918 (2018).

4. Z. Pei, Y. Yang, Q. Chen, E. Terentjev, Y. Wei, Y. Ji, Mouldable liquid-crystalline elastomer actuators with exchangeable covalent bonds. *Nat. Mater.* **13**, 36–41 (2014).

5. N. Van Zee, R. Nicolaÿ, Vitrimers: Permanently crosslinked polymers with dynamic network topology. *Prog. Polym. Sci.* **104**, 101233 (2020).

6. S. Billiet, K. De Bruycker, F. Driessen, H. Goossens, V. Speybroeck, J. Winne, F. Prez, Triazolinediones enable ultrafast and reversible click chemistry for the design of dynamic polymer systems. *Nat. Chem.* **6**, 815–821 (2014).

7. L. Chen, C. Zhao, J. Huang, J. Zhou, M. Liu, Enormous-stiffness-changing polymer networks by glass transition mediated microphase separation. *Nat. Commun.* **13**, 6821 (2022).

8. Q. Zhao, W. Zou, Y. Luo, T. Xie, Shape memory polymer network with thermally distinct elasticity and plasticity. *Sci. Adv.* **2**, e1501297 (2016).

9. B. Zhang, K. Kowsari, A. Serjouei, M. Dunn, Q. Ge, Reprocessable thermosets for sustainable three-dimensional printing. *Nat. Commun.* **9**, 1831 (2018).

10. S. Kim, M. Rahman, M. Arifuzzaman, D. Gilmer, B. Li, J. Wilt, E. Lara-Curzio, T. Saito, Closed-loop additive manufacturing of upcycled commodity plastic through dynamic cross-linking. *Sci. Adv.* **8**, eabn6006 (2022).

11. X. Zhou, Y. Zheng, H. Zhang, L. Yang, Y. Cui, B. Krishnan, S. Dong, M. Aizenberg, X. Xiong, Y. Hu, J. Aizenberg, J. Cui, Reversibly growing crosslinked polymers with programmable sizes and properties. *Nat. Commun.* **14**, 3302 (2023).

12. W. Peng, G. Zhang, Q. Zhao, T. Xie, Autonomous off-equilibrium morphing pathways of a supramolecular shape-memory polymer. *Adv. Mater.* **33**, e2102476 (2021).

13.Miao, W. Zou, B. Jin, C. Ni, N. Zheng, Q. Zhao, T. On demand shape memory polymer via light regulated topological defects in a dynamic covalent network. *Nat. Commun.* **11**, 4257 (2020).

14. C. Linghu, S. Zhang, C. Wang, K. Yu, C. Li, Y. Zeng, H. Zhu, X. Jin, Z. You, J. Song, Universal SMP gripper with massive and selective capabilities for multiscaled, arbitrarily shaped objects. *Sci. Adv.* **6**, eaay5120 (2020).

15. S. Zhuo, C. Song, Q. Rong, T. Zhao, M. Liu, Shape and stiffness memory ionogels with programmable pressure-resistance response. *Nat. Commun.* **13**, 1743 (2022).

16. Y. Yang, Z. Pei, Z. Li, Y. Wei, Y. Ji, Making and remaking dynamic 3D structures by shining light on flat liquid crystalline vitrimer films without a mold. *J. Am. Chem. Soc.* **138**, 2118–2121 (2016).

17. H. Guo, N. Sanson, A. Marcellan, D. Hourdet, Thermoresponsive toughening in LCST-type hydrogels: Comparison between semi-interpenetrated and grafted networks. *Macromolecules* **49**, 9568–9577 (2016).

18. H. Guo, N. Sanson, D. Hourdet, A. Marcellan, Thermoresponsive toughening with crack bifurcation in phase-separated hydrogels under isochoric conditions. *Adv. Mater.* **28**, 5857–5864 (2016).

19. W. Chen, L. Zhai, S. Zhang, Z. Zhao, Y. Hu, Y. Xiang, H. Liu, Z. Xu, L. Jiang, L. Wen, Cascade-heterogated biphasic gel iontronics for electronic-to-multi-ionic signal transmission. *Science* **382**, 559–565 (2023).

20. Z. Zhao, Y. Liu, K. Zhang, S. Zhuo, R. Fang, J. Zhang, L. Jiang, M. Liu, Biphasic synergistic gel materials with switchable mechanics and self-healing capacity. *Angew. Chem. Int. Ed.* **56**, 13464–13469 (2017).

21. F. Li, G. Nguyen, C. Vancaeyzeele, F. Vidal, C. Plesse, Healable ionoelastomer designed from polymeric ionic liquid and vitrimer chemistry. *ACS Appl. Polym. Mater.* **5**, 529–541 (2023).

22. N. Zheng, Z. Fang, W. Zou, Q. Zhao, T. Xie, Thermoset shape-memory polyurethane with intrinsic plasticity enabled by transcarbamoylation. *Angew. Chem. Int. Ed.* **128**, 11593–11597 (2016).

23. B. Jin, H. Song, R. Jiang, J. Song, Q. Zhao, T. Xie, Programming a crystalline shape memory polymer network with thermo- and photo-reversible bonds toward a single-component soft robot. *Sci. Adv.* **4**, eaao3865 (2018).

24. T. Kim, W. Suh, U. Jeong, Approaches to deformable physical sensors: Electronic versus iontronic. *Mater. Sci. Eng. R Rep.* **146**, 100640 (2021).

25. Y. Chang, L. Wang, R. Li, Z. Zhang, Q. Wang, J. Yang, C. Guo, T. Pan, First decade of interfacial iontronic sensing: From droplet sensors to artificial skins. *Adv. Mater.* **33**, e2003464 (2021).

26. J. Chen, J. Zhang, J. Hu, N. Luo, F. Sun, H. Venkatesan, N. Zhao, Y. Zhang, Ultrafast-response/recovery flexible piezoresistive sensors with DNA-like double helix yarns for epidermal pulse monitoring. *Adv. Mater.* **34**, e2104313 (2022).

27. X. Zhang, Z. Hu, Q. Sun, X. Liang, P. Gu, J. Huang, G. Zu, Bioinspired gradient stretchable aerogels for ultrabroad-range-response pressure-sensitive wearable electronics and high-efficient separators. *Angew. Chem. Int. Ed.* **62**, e202213952 (2023).

28. Y. Long, B. Jiang, T. Huang, Y. Liu, J. Niu, Z. L. Wang, W. Hu, Super-stretchable, anti-freezing, anti-drying organogel ionic conductor for multi-mode flexible electronics. *Adv. Funct. Mater.* **33** 2304625 (2023).

29. F. Hao, S. Wang, F. Xing, M. Li, T. Li, Y. Gu, W. Zhang, J. Zhang, Carbon-nanotube-film-based electrical impedance tomography for structural damage detection of carbon-fiber-reinforced composites. *ACS Appl. Nano Mater.* **4**, 5590–5597 (2021).

30. G. Yun, S. Tang, S. Sun, D. Yuan, Q. Zhao, L. Deng, S. Yan, H. Du, M. Dickey, W. Li, Liquid metal-filled magnetorheological elastomer with positive piezoconductivity. *Nat. Commun.* **10**, 1300 (2019).

31. Y. Cao, G. Zhang, Y. Zhang, M. Yue, Y. Chen, S. Cai, T. Xie, X. Feng, Direct fabrication of stretchable electronics on a polymer substrate with process-integrated programmable rigidity. *Adv. Funct. Mater.* **28**, 1804604 (2018).

32. R. Liu, X. Kuang, J. Deng, Y. Wang, A. Wang, W. Ding, Y. Lai, J. Chen, P. Wang, Z. Lin, H. Qi, B. Sun, Z. Wang, Shape memory polymers for body motion energy harvesting and self-powered mechanosensing. *Adv. Mater.* **30**, 201705195 (2018).

33. Q. Ze, X. Kuang, S. Wu, J. Wong, S. Montgomery, R. Zhang, J. Kovitz, F. Yang, H. Qi, R. Zhao, Magnetic shape memory polymers with integrated multifunctional shape manipulation. *Adv. Mater.* **32**, e1906657 (2020).

34. W. Liu, A. Wang, R. Yang, H. Wu, S. Shao, J. Chen, Y. Ma, Z. Li, Y. Wang, X. He, J. Li, H. Tan, Q. Fu, Water-triggered stiffening of shape-memory polyurethanes composed of hard backbone dangling PEG soft segments. *Adv. Mater.* **34**, e2201914 (2022).

35. C. Zhu, T. Bai, H. Wang, J. Ling, F. Huang, W. Hong, Q. Zheng, Z. Wu, Dual-encryption in a shape-memory hydrogel with tunable fluorescence and reconfigurable architecture. *Adv. Mater.* **33**, e2102023 (2021).

36. K. Gong, L. Hou, P. Wu, Hydrogen-bonding affords sustainable plastics with ultrahigh robustness and water-assisted arbitrarily shape engineering. *Adv. Mater.* **34**, e2201065 (2022).

37. J. Wu, Z. Zhang, Z. Wu, D. Liu, X. Yang, Y. Wang, X. Jia, X. Xu, P. Jiang, X. Wang, Strong and ultra-tough supramolecular hydrogel enabled by strain-induced microphase separation. *Adv. Funct. Mater.* **33**, 2210395 (2023).

38. T. Nonoyama, Y. Lee, K. Ota, K. Fujioka, W. Hong, J. Gong, Instant thermal switching from soft hydrogel to rigid plastics inspired by thermophile proteins. *Adv. Mater.* **32**, e1905878 (2020).

39. X. Hu, D. Zhang, S. Sheiko, Cooling-triggered shapeshifting hydrogels with multi-shape memory performance. *Adv. Mater.* **30**, e1707461 (2018).

40. Y. Zhang, W. Zhao, S. Ma, H. Liu, X. Wang, X. Zhao, B. Yu, M. Cai, F. Zhou, Modulus adaptive lubricating prototype inspired by instant muscle hardening mechanism of catfish skin. *Nat. Commun.* **13**, 377 (2022).

41. S. Zhuo, Z. Zhao, Z. Xie, Y. Hao, Y. Xu, T. Zhao, H. Li, E. Knubben, L. Wen, L. Jiang, M. Liu, Complex multiphase organohydrogels with programmable mechanics toward adaptive soft-matter machines. *Sci. Adv.* **6**, eaax1464 (2020).

42. Z. Zhao, S. Zhuo, R. Fang, L. Zhang, X. Zhou, Y. Xu, J. Zhang, Z. Dong, L. Jiang, M. Liu, Dual-programmable shape-morphing and self-healing organohydrogels through orthogonal supramolecular heteronetworks. *Adv. Mater.* **30**, e1804435 (2018).

43. J. Park, J. Sun, Phase-transitional ionogel-based supercapacitors for a selective operation. *ACS Appl. Mater. Interfaces* **14**, 23375–23382 (2022).

44. X. Ming, L. Yao, H. Zhu, Q. Zhang, S. Zhu, Dramatic and reversible water-induced stiffening driven by phase separation within polymer gels. *Adv. Funct. Mater.* **32**, 2109850 (2022).

45. T. Ho, B. Febriansyah, N. Yantara, S. Pethe, D. Accoto, S. Pullarkat, N. Mathews, Inducing thermoreversible optical transitions in urethane-acrylate systemsviaionic liquid incorporation for stretchable smart devices. *J. Mater. Chem. A* **9**, 13615–13624 (2021).

46. X. Ming, L. Shi, H. Zhu, Q. Zhang, Stretchable, phase-transformable ionogels with reversible ionic conductor-insulator transition. *Adv. Funct. Mater.* **30**, 2005079 (2020).

47. S. Xi, F. Tian, G. Wei, X. He, Y. Shang, Y. Ju, W. Li, Q. Lu, Q. Wang, Reversible dendritic-crystal-reinforced polymer gel for bioinspired adaptable adhesive. *Adv. Mater.* **33**, e2103174 (2021).

48. Y. Kamiyama, R. Tamate, T. Hiroi, S. Samitsu, K. Fujii, T. Ueki, Highly stretchable and self-healable polymer gels from physical entanglements of ultrahigh-molecular weight polymers. *Sci. Adv.* **8**, eadd0226 (2022).

49. R. Tamate, K. Hashimoto, T. Horii, M. Hirasawa, X. Li, M. Shibayama, M. Watanabe, Self-healing micellar ion gels based on multiple hydrogen bonding. *Adv. Mater.* **30**, e1802792 (2018).

50. A. Legrand, C. Soulié-Ziakovic, Silica-epoxy vitrimer nanocomposites. *Macromolecules* **49**, 5893–5902 (2016).

51. C. Zhang, B. Jin, X. Cao, Z. Chen, W. Miao, X. Yang, Y. Luo, T. Li, T. Xie, Dielectric polymer with designable large motion under low electric field. *Adv. Mater.* **34**, e2206393 (2022).

52. L. Odenwald, F. Wimmer, N. Mast, M. Schußmann, M. Wilhelm, S. Mecking, Molecularly defined polyolefin vitrimers from catalytic insertion polymerization. *J. Am. Chem. Soc.* **144**, 13226–13233 (2022).

53. J. Zhao, Z. Zhang, L. Cheng, R. Bai, D. Zhao, Y. Wang, W. Yu, X. Yan, Mechanically interlocked vitrimers. *J. Am. Chem. Soc.* **144**, 872–882 (2022).

54. C. He, S. Shi, D. Wang, B. Helms, T. Russell, Poly (oxime-ester) vitrimers with catalyst-free bond exchange. *J. Am. Chem. Soc.* **141**, 13753–13757 (2019).

55. M. Guerre, C. Taplan, R. Nicolaÿ, J. Winne, F. Prez, Fluorinated vitrimer elastomers with a dual temperature response. *J. Am. Chem. Soc.* **140**, 13272–13284 (2018).

56. C. Tretbar, J. Neal, Z. Guan, Direct silyl ether metathesis for vitrimers with exceptional thermal stability. *J. Am. Chem. Soc.* **141**, 16595–16599 (2019).

57. W. Denissen, G. Rivero, R. Nicolaÿ, L. Leibler, J. Winne, F. Prez, Vinylogous urethane vitrimers. *Adv. Funct. Mater.* **25**, 2451–2457 (2015).

58. Z. Hu, F. Hu, L. Deng, Y. Yang, Q. Xie, Z. Gao, C. Pan, Y. Jin, J. Tang, G. Yu, W. Zhang, Reprocessible triketoenamine based vitrimers with closed-loop recyclability. *Angew. Chem. Int. Ed.* **62**, e202306039 (2023).

59. L. Yue, Y. Su, M. Li, L. Yu, S. Montgomery, X. Sun, M. Finn, W. Gutekunst, R. Ramprasad, H. Qi, One-pot synthesis of depolymerizableδ-lactone based vitrimers. *Adv. Mater.* **35**, e2300954 (2023).

60. M. Wang, P. Zhang, M. Shamsi, J. Thelen, W. Qian, V. Truong, J. Ma, J. Hu, M. Dickey, Tough and stretchable ionogels by in situ phase separation. *Nat. Mater.* **21**, 359–365 (2022).

61. V. Amoli, J. Kim, E. Jee, Y. Chung, S. Kim, J. Koo, H. Choi, Y. Kim, D. Kim, A bioinspired hydrogen bond-triggered ultrasensitive ionic mechanoreceptor skin. *Nat. Commun.* **10**, 4019 (2019).

62. D. Kim, X. Liu, B. Yu, S. Mateti, L. Dell, Q. Rong, Y. Chen, Amine-functionalized boron nitride nanosheets: A new functional additive for robust, flexible ion gel electrolyte with high lithium-ion transference number. *Adv. Funct. Mater.* **30**, 1910813 (2020).

63. H. Lee, A. Erwin, M. Buxton, M. Kim, A. Stryutsky, V. Shevchenko, A. Sokolov, V. Tsukruk, Shape persistent, highly conductive ionogels from ionic liquids reinforced with cellulose nanocrystal network. *Adv. Funct. Mater.* **31**, 2103083 (2021).

64. L. Yu, S. Guo, Y. Lu, Y. Li, X. Lan, D. Wu, R. Li, S. Wu, X. Hu, Highly tough, Li-metal compatible organic-inorganic double-network solvate ionogel. *Adv. Energy Mater.* **9**, 1900257 (2019).

65. J. Zhang, J. Yin, N. Li, H. Liu, Z. Wu, Y. Liu, T. Jiao, Z. Qin, Simultaneously enhancing the mechanical strength and ionic conductivity of stretchable ionogels enabled by polymerization-induced phase separation. *Macromolecules* **55**, 10950–10959 (2022).

66. M. Zhang, R. Yu, X. Tao, Y. He, X. Li, F. Tian, X. Chen, W. Huang, Mechanically robust and highly conductive ionogels for soft ionotronics. *Adv. Funct. Mater.* **33**, 2208083 (2023).

67. X. Yu, Y. Zheng, Y. Wang, H. Zhang, H. Song, Z. Li, X. Fan, T. Liu, Facile fabrication of highly stretchable, stable, and self-healing ion-conductive sensors for monitoring human motions. *Chem. Mater.* **34**, 1110–1120 (2022).

68. W. Li, L. Li, S. Zheng, Z. Liu, X. Zou, Z. Sun, J. Guo, F. Yan, Recyclable, healable, and tough ionogels insensitive to crack propagation. *Adv. Mater.* **34**, e2203049 (2022).

69. L. Li, W. Li, X. Wang, X. Zou, S. Zheng, Z. Liu, Q. Li, Q. Xia, F. Yan, Ultra-tough and recyclable ionogels constructed by coordinated supramolecular solvents. *Angew. Chem. Int. Ed.* **61**, e202212512 (2022).

70. P. Zhang, I. Lei, G. Chen, J. Lin, X. Chen, J. Zhang, C. Cai, X. Liang, J. Liu, Integrated 3D printing of flexible electroluminescent devices and soft robots. *Nat. Commun.* **13**, 4775 (2022).

71. K. Cho, S. An, D. Cho, J. Kim, J. Nam, M. Kim, K. Lee, Block copolymer-based supramolecular ionogels for accurate on-skin motion monitoring. *Adv. Funct. Mater.* **31**, 2102386 (2021).

72. Z. Yu, P. Wu, Underwater communication and optical camouflage ionogels. *Adv. Mater.* **33**, e2008479 (2021).

73. M. Yao, B. Wu, X. Feng, S. Sun, P. Wu, A highly robust ionotronic fiber with unprecedented mechanomodulation of ionic conduction. *Adv. Mater.* **33**, e2103755 (2021).

74. L. Xu, Z. Huang, Z. Deng, Z. Du, T. L. Sun, Z. Guo, K. Yue, A transparent, highly stretchable, solvent-resistant, recyclable multifunctional ionogel with underwater self-healing and adhesion for reliable strain sensors. *Adv. Mater.* **33**, e2105306 (2021).

75. W. Li, L. Li, Z. Liu, S. Zheng, Q. Li, F. Yan, Supramolecular ionogels tougher than metals. *Adv. Mater.* **35**, e2301383 (2023).

76. Z. Cao, H. Liu, L. Jiang, Transparent, mechanically robust, and ultrastable ionogels enabled by hydrogen bonding between elastomers and ionic liquids. *Mater. Horiz.* **7**, 912–918 (2020).

77. Y. Ren, J. Guo, Z. Liu, Z. Sun, Y. Wu, L. Liu, F. Yan, Ionic liquid-based click-ionogels. *Sci. Adv.* **5**, eaax0648 (2019).

78. H. Dinh Xuan, B. Timothy, H. Park, T. Lam, D. Kim, Y. Go, J. Kim, Y. Lee, S. Ahn, S Jin, J. Yoon, Super stretchable and durable electroluminescent devices based on double-network ionogels. *Adv. Mater.* **33**, e2008849 (2021).

79. X. Han, Z. Lv, F. Ran, L. Dai, C. Li, C. Si, Green and stable piezoresistive pressure sensor based on lignin-silver hybrid nanoparticles/polyvinyl alcohol hydrogel. *Int. J. Biol. Macromol.* **176**, 78–86 (2021).

80. G. Mogli, A. Chiappone, A. Sacco, C. Pirri, S. Stassi, Ultrasensitive piezoresistive and piezocapacitive cellulose-based ionic hydrogels for wearable multifunctional sensing. *ACS Appl. Electron. Mater.* **5**, 205–215 (2023).

81. S. Park, G. Kim, Piezo-impedance response of carbon nanotube/polydimethylsiloxane nanocomposites. *APL Mater.* **7**, 041118 (2019).

82. J. Teixeira, L. Horta-romarís, M. Abad, P. Costa, S. Lanceros-méndez, Piezoresistive response of extruded polyaniline/(styrene-butadiene-styrene) polymer blends for force and deformation sensors. *Mater. Des.* **141**, 1–8 (2018).

83. G. Yun, S. Tang, H. Lu, T. Cole, S. Sun, J. Shu, J. Zheng, Q. Zhang, S. Zhang, M. Dickey, W. Li, Liquid metal hybrid composites with high-sensitivity and large dynamic range enabled by micro- and macrostructure engineering. *ACS Appl. Polym. Mater.* **3**, 5302–5315 (2021).

84. H. Liu, Y. Li, M. Zhou, B. Chen, Y. Chen, W. Zhai, Ambilateral convergent directional freeze casting meta-structured foams with a negative Poisson’s ratio for high-performance piezoresistive sensors. *Chem. Eng. J.* **454**, 140436 (2023).

85. G. Yun, S. Tang, Q. Zhao, L. Deng, M. D. Dickey, W. Li, G. Yun, S. Tang, Q. Zhao, Y. Zhang, H. Lu, D. Yuan, Liquid metal composites with anisotropic and unconventional piezoconductivity. *Matter* **3**, 824–841 (2020).
